# Facebook and mosquito-borne disease outbreaks: An analysis of public responses to federal health agencies’ posts about dengue and Zika in 2016

**DOI:** 10.1371/journal.pgph.0000977

**Published:** 2022-09-12

**Authors:** Pablo Carvajal, Jo Anne G. Balanay, Sachiyo Shearman, Stephanie L. Richards

**Affiliations:** 1 Department of Health Education and Promotion, East Carolina University, Greenville, North Carolina, United States of America; 2 School of Communication, East Carolina University, Greenville, North Carolina, United States of America; University of Oslo Faculty of Medicine: Universitetet i Oslo Det medisinske fakultet, NORWAY

## Abstract

Responses of Facebook users to four United States federal health agencies’ social media posts about dengue and Zika (mosquito-borne diseases), surveillance, and control during the Zika outbreak in 2016 were tracked. Official Facebook pages of health agencies were analyzed, and a qualitative analysis program was used to perform a thematic analysis of the data on public responses to health agency posts. Public sentiment analysis showed that Facebook users had a negative sentiment towards health information observed in this study. Themes were identified in the studied posts, giving insight into the nature of public discussions and responses to federal health agencies. Themes were assessed based on the way the agencies’ mosquito-borne disease information was received by the public through the social media platform, Facebook. Results indicate that public perception/understanding of mosquito-borne disease outbreaks can be assessed by analyzing public interactions with health agencies on Facebook. The importance of maximizing effectiveness by addressing issues in sharing health education information, risk communication, and monitoring of public responses by health agencies through social media platforms is discussed.

## Introduction

Vector-borne diseases are responsible for significant global morbidity and mortality, particularly impacting the most disadvantaged populations in the developing world [1, 2]. It is important to establish mosquito control programs (MCPs) that rely on public risk communication/education and surveillance-based targeted control (e.g., insecticide application and other methods). Communication with the communities served by MCPs can help sustain these programs over time by garnering public support. Continued global public health issues with vector-borne diseases like dengue, and Zika necessitate concerted efforts to establish and maintain effective MCPs. Nearly half the global population lives in areas at risk for dengue [3] and Zika. The primary vectors of both dengue virus (DENV) and Zika virus (ZIKV), pathogens that respectively cause dengue and Zika, are *Aedes aegypti* Linnaeus (blood feeds primarily on humans) and *Aedes albopictus* Skuse. Although the primary vectors of ZIKV are these *Aedes* mosquitoes, this virus differs from DENV in that sexual transmission also contributes to its spread [4]. The significant increase in dengue cases over the past five decades is due to various factors, including increased urbanization, internal and international migration, inconsistent water supplies (creating mosquito oviposition sites), and ineffective or absent MCPs [5].

Mosquito control programs should be involved with communicating public health information to help prevent vector-borne diseases [6]. In the United States (US), MCPs that conduct public education, mosquito and pathogen surveillance, site assessments, and insecticide applications are not very common (largely due to funding constraints) [7, 8], as only about half of the approximately 2,800 local health departments offer these services [6]. An example of how lack of funding (leading to fewer mosquito control experts) can impact the effectiveness of MCPs in North Carolina (NC) is the disbandment of its Public Health Pest Management section within NC Department of Environmental and Natural Resources. This resulted in underfunded/absent MCPs across NC, leading to increased risk of disease due to a lack of continuous surveillance and control [9]. Local MCP efforts can be complemented by state (e.g., Division of Public Health) and federal (e.g., Centers for Disease Control and Prevention [CDC]) health campaigns, often promoted and disseminated through the media [10].

Published studies assessing efficacy of control interventions and public health education in relation to prevention of disease are limited. Others studied public behavior and attitudes of US populations during news coverage (August 2015—April 2016) on the first human trial of the ZIKV vaccine [11]. The same study indicated that news coverage of the ZIKV vaccine resulted in a short-term increase in public confidence in science, suggesting that confidence may improve with regular science-based media coverage about scientific advances.

Media (e.g., television/radio/internet news) coverage of previous mosquito-borne disease outbreaks and the public’s reaction to this information may give insight into development of future public health education and risk communication campaigns. Social media platforms (e.g., Facebook, Instagram, Twitter) can play an important role in delivering information (accurate or inaccurate) during an outbreak [12]. Public health organizations like the CDC use media platforms such as Facebook to deliver pertinent information (e.g., symptoms, methods for prevention) about health threats, such as dengue and Zika. Since some view social media outlets like Facebook or Twitter as a source of reliable health information [13], it is important to consider the quality and delivery of health information and public reactions to that information. A common unnamed theory about the role of media coverage in health education is that, once the number of cases of an emerging disease increase (thereby receiving more media coverage), the number of infections may start to decline due to increased public awareness and associated preventative behaviors [14, 15]. Others found that, as media reports about the 2009 H1N1 (influenza A) pandemic increased, more individuals increased preventative behaviors (e.g., social distancing, vaccination) [14]. However, findings indicate that media fatigue and reversal of preventative behaviors can reduce the positive benefits of interventions via social media [14].

The media can educate the public about emerging health issues, health trends, and solutions. Social media platforms (e.g., Facebook, Twitter) can be a hub for specialized information, and there are even outlets (e.g., Twitter handles) that focus on news, alerts, and/or information about *Ae*. *aegypti* and associated diseases [16]. A New York Times article reported that misinformation (e.g., messages indicating the vaccine was dangerous or a scam) regarding yellow fever virus (transmitted by *Ae*. *aegypti*) negatively impacted a vaccination campaign for yellow fever in Brazil [17]. Misinformation rapidly spread through social media and encouraged public mistrust of a government health campaign that aimed to vaccinate more than 23 million people in areas of Rio de Janeiro, Brazil [17]. While media outlets can be helpful in disease prevention strategies, complementing MCPs by offering health education and encouraging public support, media sources can also be used to spread misinformation [12].

Public health officials must use proper science-based risk communication tools to effectively communicate risk to the public [18–20]. Control methods directed against *Ae*. *aegypti* and *Ae*. *albopictus*, such as source reduction, larvicides (e.g., *Baccillus thuringiensis*, insect growth regulators), and introduction of natural predators have shown mixed success rates due, in part, to lack of community involvement and awareness [21]. When organized and planned properly to communicate science-based health information, media outlets could encourage community involvement by disseminating information about mosquito-borne diseases to large numbers of people. Media platforms can encourage public participation through open forums, where relevant health information can be delivered and where questions about potential public health threats can be quickly answered. In 2016, the CDC hosted a live chat on Twitter to address public concerns about ZIKV in the US [22]. In 2015, the CDC hosted a similar session about Ebola virus; although this is not a mosquito-borne disease, the forum provided timely information about disease symptoms, modes of transmission, and methods of prevention [23]. An analysis of Facebook posts during January-June 2016 identified 200 of the most popular posts (most [N = 121/200] from news agencies) about Zika (81% of these posts were shown to have credible information) [24]. During this period, the same study showed the CDC had *ca*. 22 posts with *ca*. 1,000 shares. Traditional media (e.g., websites, newspapers, television, radio, flyers) has been used to provide information about dengue [25–27]. Media outlets are important tools for public education and the media is the primary source of information on dengue for most people [25–27].

Television and other modern forms of media (e.g., social media) should be utilized as part of local MCPs’ connection to the public. Media tools and platforms likely impact the dissemination of information through personal contacts like family, neighbors, and friends. In the US, > 90% of households have access to television [28]. Any media source can spread misinformation. For example, during a yellow fever public education campaign, people used the WhatsApp messaging service to disseminate rumors (i.e., videos, graphics, and texts) that the yellow fever vaccine was ineffective [17]. The same study indicated that WhatsApp "is a fundamental characteristic of the way we circulate information, news, etc.," in Brazil. Since WhatsApp messages come from known contacts, recipients may value the shared content to a greater extent without verifying validity [17]. Analysis of Facebook posts during the first six months of the Zika outbreak in 2016 showed *ca*. 21/200 (16%) of posts were misleading (e.g., Zika was going to kill a large number of individuals in third world countries or that the outbreak was not real and related to covering up potential dangers of pesticides) [24].

Using social media for public health education about mosquito control and exposure prevention strategies (e.g., using insecticide-treated bednets, source reduction) could strengthen community-based environmental management [26]. Educating the public about mosquito control through social and traditional media outlets (e.g., television, newspaper) has shown promising results in reducing vector propagation in rural communities in Malaysia [25]. Although media coverage of emerging infectious diseases can be sporadic, rather than comprehensive and consistent [29], its use by public health agencies for risk communication may help disseminate information and garner public attention–a key opportunity to educate the public on the importance of disease prevention and control. Online search behavior and social media interactions are reactive to media coverage, especially for new and concerning public health threats [29].

The current study analyzed social media coverage and public responses to federal health agencies’ posts on US mosquito-borne disease outbreaks (i.e., Zika, dengue). Recommendations on how public health education and risk communication via social media can be improved are discussed. Media coverage and public health campaigns for Zika and dengue outbreaks in the US were analyzed. We aimed to: 1) Identify and describe the delivery of mosquito-borne disease prevention information by federal health agencies through Facebook during a Zika outbreak, 2) Analyze public responses to federal health agency social media posts about mosquito-borne disease; 3) Analyze federal health agency posts using the Crisis and Emergency Risk Communication model (CERC), and 4) Provide recommendations to improve public health educational campaigns about mosquito-borne diseases using social media.

## Materials and methods

### 1. Coding materials

A mixed methods approach was used, including descriptive statistics and quantitative data analysis. Public perception of information delivered by federal health agencies in the US was analyzed with respect to Zika and dengue. Data were used to evaluate whether the public trusts health agencies as reliable sources of health information on the internet (e.g., social media). This study focused on data from the CDC, US Food and Drug Administration (FDA), National Institutes of Allergy and Infectious Diseases (NIAID), and the National Institutes of Health (NIH). Information was collected from the agencies’ respective Facebook pages for January-December 2016. This period was selected because the CDC issued an alert for American travelers in January 2016, and because January-December 2016 was when a ZIKV outbreak occurred in Miami, Florida [30]. Facebook was used as the source of publicly available data due to the relevancy and utility of this platform, given that more than 64% of US adults are Facebook users, and half of these users repeatedly get their news from this source [31].

#### 1a. Data and ethics statement

Data collection and analysis methods complied with the terms and conditions for the source of the data.The East Carolina University Institutional Review Board (IRB) determined that information was collected from social media pages that are publicly available (they can be reached from a simple web search). Further, the information consisted of comments on the social media pages of several federal agencies (public comments with no reasonable expectation of privacy). As such, the data used in this research would not be considered private information nor would it constitute human subjects research. As it was not human subjects research, it does not fall under the purview of the IRB or require IRB approval.

#### 1b. Data selection and organization process

NVivo’s NCapture version 1.5.1 [32] data gathering tool was used to collect social media and other types of posts (e.g., news releases, event announcements, infographics) from the agencies related to Zika and dengue. The content of health agencies’ posts, along with corresponding public responses, were organized, analyzed, and coded for keywords, themes, sentiments, and common expressions. Coding established identifiers (e.g., keyword, theme, sentiment) that were compared with each post’s content to detect presence and analyze the content. Initial coding was performed by investigators whereas a list of keywords, concepts, and sentiments was gathered. Investigators created a coding list that was later integrated into NVivo. An automated tool in NVivo that performs a similar process (i.e., coding for a list of keywords, sentiments, and themes, based on analysis of the gathered data by NCapture) was also executed and reviewed by investigators for accuracy. Both datasets were reviewed for accuracy and relevance to dengue and Zika information sharing and public sentiment before being imported into NVivo.

#### 1c. Coding data keywords and quarterly period of analysis

Further coding was performed to identify common concepts and ideas, summarized into individual codes (e.g., “conspiracy”, “Zika”, “bite”, “fake”) and common/interrelated codes (i.e., themes in NVivo). Codes and themes were used to analyze subsequent posts. A parent theme (e.g., “birth defects”) may also have one or more daughter codes (e.g., “heart defects”, “terrible birth defects”, “low birth weight”) that describe the parent theme in more detail. Here, coding was based on thematic analysis, a qualitative analytic method used to analyze data by themes, since the analysis required interpretation of the content of media posts, and the assessed reaction of public respondents to those posts. Themes were automatically created with NVivo and later analyzed/modified by investigators. Content was removed if words and/or sentences were irrelevant to our search terms related to ZIKV or DENV. Negative and positive sentiments within each theme were determined by NVivo and manually verified by investigators for accuracy.

Facebook pages for CDC, FDA, NIAID, and NIH were evaluated for information posted between January-December 2016. Data were divided by quarter to compare the frequency of official arboviral information posted as well as user interactions throughout the time frame (i.e., quarters; 1^st^: January-March; 2^nd^: April-June; 3^rd^: July-September; 4^th^: October-December 2016). These federal health agencies were selected because they offered the most search results and content most relevant to Zika and dengue after performing key word searches, using the NCapture feature of the NVivo program. The following search terms were initially used to assess titles and other posted content (results in NCapture) in Facebook:

(i) [Zika OR dengue OR mosquito-borne disease OR epidemic OR outbreak OR risk OR microcephaly OR virus]

AND

(ii) [misinformation OR fake news OR disinformation OR rumo* OR false OR mislead* spread OR propagate* OR disseminat* OR circulat* OR communicat* OR diffuse OR broadcast OR inform* health OR disease OR infectious OR virus OR vaccin* OR dengue OR Zika OR aedes OR false OR fake OR conspiracy OR mosquito borne disease OR epidemic OR outbreak OR risk OR microcephaly OR virus]

The initial search led to the selection of these four federal health agencies as the target Facebook pages from which data would be gathered. Important themes related to ZIKV and DENV discussed among Facebook users were identified. Keywords (eliminating common language words not related to the subject from our query runs in NVivo; i.e., articles, prepositions, and conjunctions) were then identified. Keywords and their frequencies from posts were used to build word clouds to visualize important themes and their progression throughout the study period in 2016. Posts were organized by publication date and divided into four quarters for 2016, resulting in four-word clouds and bar graphs for data display.

### 2. Coding procedures

Thematic analysis followed trends in previous studies of social media public health information dissemination and public perception of disease outbreaks [33–35]. Themes and subthemes were created based on analysis of the data in NVivo, and the number of posts associated with each theme were determined. Extracted items (i.e., keywords from posts) were plotted into word clouds to assess the extent to which legitimate information and/or misinformation was reported, as well as public perception.

#### 2a. Sentiment analysis

Data were sorted by keywords, themes, sentiments, and common expressions. Data were organized by sentiments (e.g., positive, negative) based on perceived respondent sentiment towards a post’s information and were further sub-divided as “very positive”, “moderately positive”, “very negative” and “moderately negative” before being graphed (as percent positive or negative). In cases where conflicting/refuted information and/or discrediting of content occurred within posts, sentiments were ranked as very negative. Criteria to establish a sentiment (negative vs. positive) were based on the prevalence of codes, common expressions, and themes associated with a sentiment and determining which type of sentiment was dominant in each post. Sentiment assessments of each post were performed by investigators and then compared to results of the automated tool in NVivo that analyzed sentiment by analyzing content based on a post’s codes, identified themes, grammar, and sentence structure. A set of 98 coded terms was created to analyze the posts, discover their frequency and relevancy, and guide further sentiment analyses (Tables A-D in S1 Text). Themes were automatically created with NVivo which identified themes commonly occurring in each set of the previously identified posts by quarter. The list of subordinate codes was larger, with a total number of 953 possible codes created and these were coded as single words or as a set of words. An example of a parent theme and subordinate codes can be found in Table C in S1 Text. Coding helped with the interpretation of sentiments and/or perception of Facebook posts. Sentences from posts were analyzed to assess the sentiment towards the subject of the posts, and relevancy to the posts’ objective. Only sentiments specific and relevant to our search terms related to Zika and dengue were captured, hence spam-type posts and other unrelated posts were not analyzed here.

#### 2b. Thematic analysis

Themes from the CERC were adapted and applied here to further evaluate the data [36]. The CERC is a CDC program that relies on past public health emergencies and research in the fields of public health, psychology, and emergency risk communication. The CERC outlines strategies across a series of crisis phases and provides guidance on social media public health communication [36]. Potential crisis phases are categorized as risk, eruption, clean-up, recovery, and evaluation [36]. Knowledge of crisis phases and timelines help emergency managers anticipate communication needs [36].

Themes adapted from CERC and used here include: 1) risk messages: posts with information about Zika and dengue, mosquitoes and how arboviruses are transmitted, and disease symptoms; 2) warnings: posts about risk factors/dangers related to Zika and dengue; 3) preparation guidance: posts mentioning health agencies responding to the diseases and recommendations on how to respond; 4) reducing uncertainty: posts with information about cases, local areas impacted, and sources of information; 5) increasing efficacy: posts about personal prevention measures and expressions about shared responsibility for disease prevention; and 6) reassurance: posts that comfort the public by mentioning specific government action, and that recognize the public’s efforts in preventing and fighting the diseases [36].

## Results

A total of 252 posts were collected during the search process from across the four federal health agencies studied here (CDC, FDA, NIAID, and NIH). Several (n = 38) posts were excluded because of irrelevant or inadequate content related to ZIKV or DENV, hence we collected 214 relevant posts (ZIKV = 198 posts; DENV = 16 posts). Most posts were from the CDC (n = 94 posts), followed by the FDA (n = 78 posts), NIAID (n = 27 posts), and NIH (n = 15 posts).

Posts were plotted (based on similar words and themes) and lumped together into more general “codes” or single words, and then word clouds were generated to assess the extent to which legitimate information and/or misinformation was reported. Public perception of information was also analyzed for Facebook posts of federal health agencies. Some posts by government agencies were characterized as misinformation, or as “fake” or “conspiracy” by some respondents to these agencies’ Facebook posts. Some information offered by the same respondents (i.e., the information within some Facebook user’s response comments) to the agency posts, often offered conflicting and/or inaccurate information.

**Fig 1:**
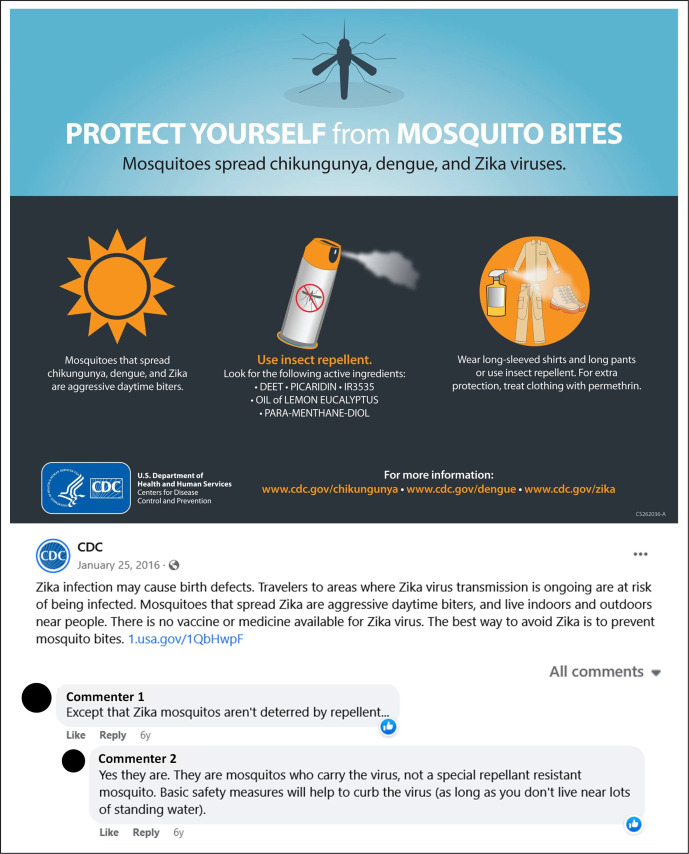
Centers for Disease Control and Prevention Facebook post about mosquito-borne diseases. Source: CDC; Materials developed by CDC). Reference to specific commercial products, manufacturers, companies, or trademarks does not constitute its endorsement or recommendation by the US Government, Department of Health and Human Services, or CDC. Unless a copyright is indicated, information on CDC’s sites, social media profiles, blogs, and applications are in the public domain and may be copied and distributed without permission.

Fig 1 demonstrates an example of one of the CDC’s first (January 2016) Facebook posts about Zika and other mosquito-borne diseases. The publication uses both text and imagery to convey a message primarily about ZIKV, its transmission, the lack of vaccine treatment, and prevention methods. The post allows for public reaction and allows the public to respond to the message through “Comments” and “Likes”—a public comment is included in this figure, showing an instance of the public providing information that is contradictory or misleading (i.e., respondent comment: “Except that Zika mosquitoes aren’t deterred by repellent”).

### Keyword analysis

Word clouds were created by plotting the 50 most frequently used words and themes found in the Facebook posts by quarter (1^st^: January-March; 2^nd^: April-June; 3^rd^: July-September; 4^th^: October-December 2016). This visual analysis shows the nature of information published by all four government agencies and associated public reaction.

**Fig 2:**
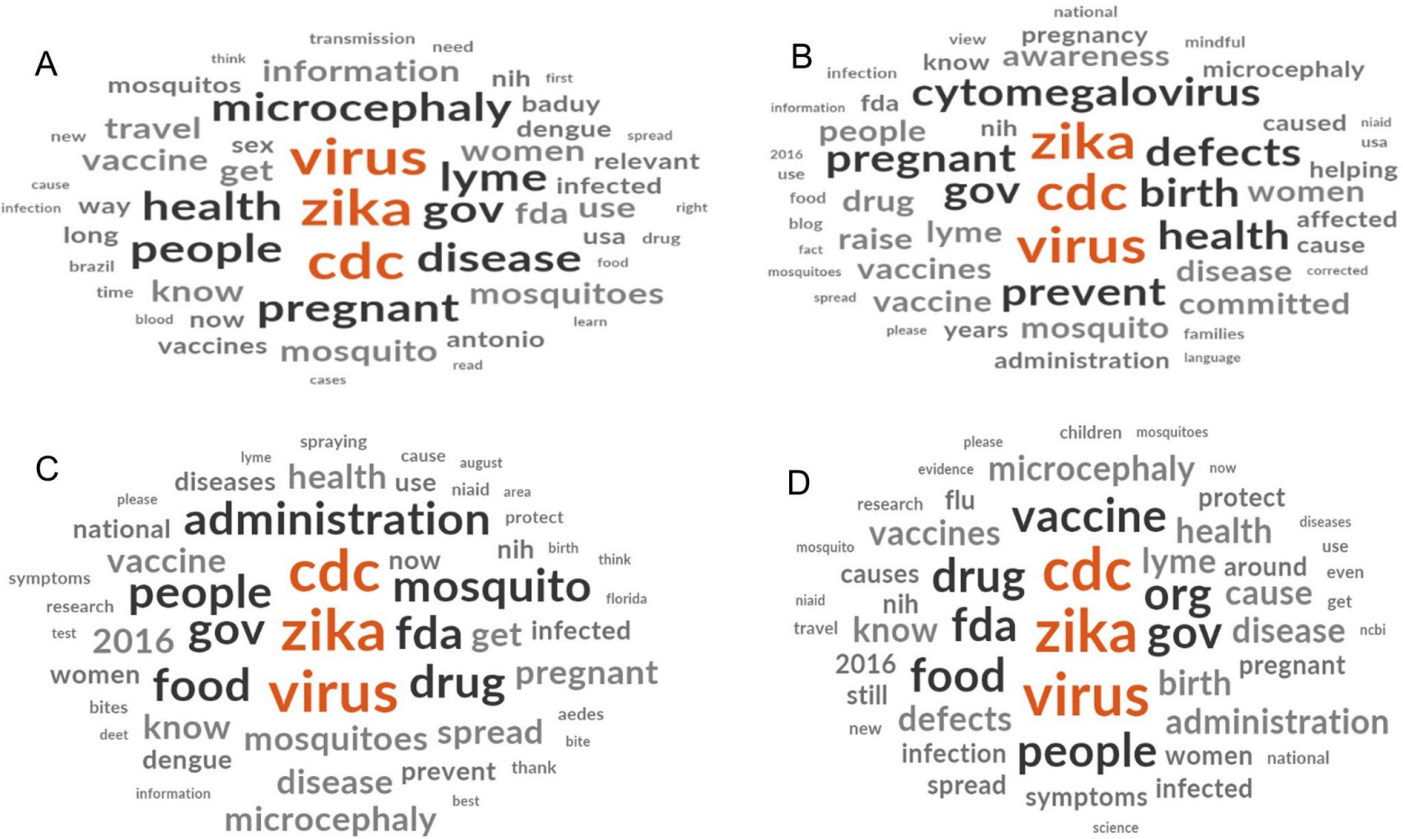
Word cloud with prevalent keywords on Facebook posts for A) January-March 2016, B) April-June 2016, C) July-September 2016, D) October-December 2016.

Fig 2A shows prevalent keywords during January-March 2016. This quarter showed the least number of posts (n = 35; most posts in February), likely due to initial ZIKV incidence/outbreaks being concentrated in South America (i.e., Brazil) and some Caribbean areas. Some posts during this quarter focused on international travel and how events (e.g., Olympics and Carnival [January in Brazil]) could have been affected by the ZIKV outbreak. Some posts (infrequently) mentioned the transmission cycle of ZIKV and/or DENV in general. Impacts of ZIKV on pregnancy (e.g., microcephaly) were major themes during this period.

Fig 2B shows prevalent keywords for April-June 2016. This quarter showed an increased number of posts (n = 44; *ca*. 50% in June). This increase may reflect the rapid spread of ZIKV to regions outside of Brazil, and the US government’s increased warnings to traveling US citizens. Some of the same themes are discussed as in the previous quarter but additional keywords began to appear, most notably those that describe women’s health (e.g., pregnant) and symptoms of the virus (e.g., cytomegalovirus [congenital infection]).

Fig 2C shows the most frequently occurring keywords and themes for July-September 2016. This quarter showed an increase in frequency of posts (n = 95; 40% in August), signaling what may have been the height of social media communication by the US agencies studied here. There was a 171% increase in the number of posts in this quarter compared to the first quarter. The nature of keywords during this time frame were related to government actions to protect citizens (e.g., administration, people, drug). At this point, ZIKV was being transmitted in US areas of Florida and Puerto Rico.

Fig 2D shows the most common keywords during October-December 2016. This quarter had a similar number of posts (n = 44) as the second quarter. The wane in communication by the studied government agencies suggests a decrease in ZIKV relevancy to the US public, perhaps as information had already been widely disseminated in the prior months. Keywords during this quarter continue to show the importance of treatments (e.g., vaccine, drug).

### Sentiment analysis

**Fig 3:**
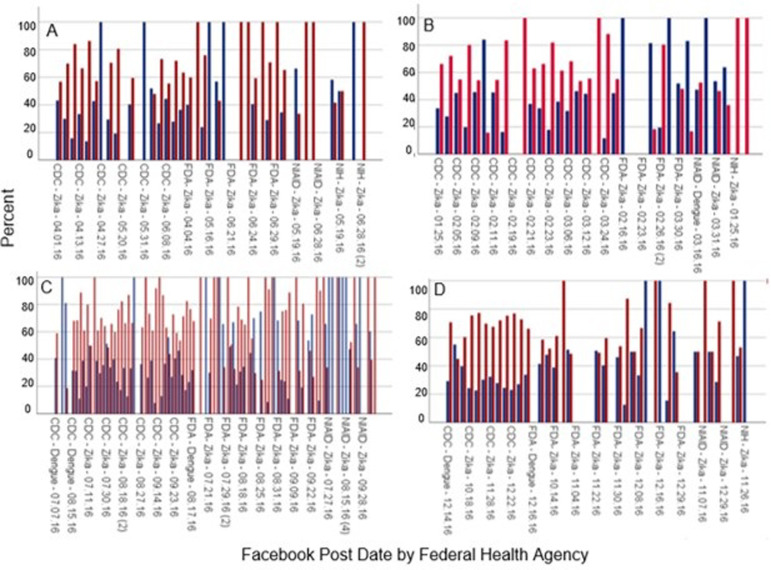
Percentage of sentiment based on coding from A) Quarter 1; January-March 2016, B) Quarter 2; April-June 2016, C) Quarter 3; July-September 2016, D) Quarter 4; October-December 2016. The date on the x-axis represents the date of a post in first quarter of the year 2016. Abbreviations: Centers for Disease Control and Prevention (CDC); Food and Drug Agency (FDA); National Institutes of Allergy and Infectious Disease (NIAID); National Institutes of Health (NIH).

The sentiment (i.e., positive or negative tone) indicated that posts about ZIKV clearly overshadowed those about DENV (Fig 3). Table A in S1 Text shows “references” as the number of times the code was found in the files (number of posts) per quarter. A set of themes was also created so that codes could be grouped by theme (i.e., 113 “parent” themes were created) (Table B in S1 Text). Of the total 214 posts studied, a mixture of negative (52%) and positive (48%) public feedback was observed about information posted by the studied federal health agencies. An example of this is that a negative sentiment would be deduced from the following user excerpts on a post about postponing travel to the Miami-Dade area due to ZIKV transmission in the area (post published by the CDC on 10/19/2016). The following are three user excerpts in response to the CDC post: User 1 exerpt from 10/25/2016 expressing skepticism about the causes of clinical outcomes related to ZIKV: “How it is that so many individual doctors are allowing this shrunken brain epidemic to be laid on Zika? Is there no attending physician anywhere who has another insight into the sudden onset of this unfortunate malady…they are now fronting brain damage caused by a newly formulated Tdap vaccine as a Zika virus outbreak.” User 2 exerpt from 10/25/2016 questioning CDC advice on the topic: “So the CDC believes that it’s outlandish to tell pregnant women to kiss their toddlers on the forehead (CMV) but you can tell them to avoid entire neighborhoods (Zika)?”; User 3 excerpt from 10/19/2016 describing their feelings: “Scary”. In Fig 3, the negative sentiment percentages are colored in red. Tables E, F in S1 Text demonstrate the number of codes associated with a negative or positive sentiment found in each individual post studied. Table F in S1 Text also provides percentages of overall positive or negative feedback for each post. The data show that most people had a negative response to the information from government social media posts, with increasingly negative reception to the information as coverage of the pandemic progressed.

Quarter 1 data were all related to ZIKV (no DENV) and show that 57% and 43% of the posts in this timeframe received negative and positive feedback, respectively (Fig 3A). Limited information on ZIKV or DENV was available from the government agencies studied here prior to January 2016. The increase in social media (Facebook) posts after January 2016 can be attributed to the global spread of ZIKV. Information from posts about ZIKV and DENV were received with positive sentiment at the beginning of the ZIKV epidemic, with an increasing negative sentiment towards the information published by the CDC, NIAID, NIH, and the FDA in the third and final quarters analyzed in this study. When assessing Quarter 2, data show that 62% and 38% of the posts in this timeframe received negative and positive feedback, respectively (Fig 3B).

In January 2016, the CDC had issued an alert for people traveling to places where ZIKV outbreaks were occurring. It is likely that the US public would have been eager to learn ZIKV information after the CDC’s health alert. The first cases of ZIKV reported in the US occurred in Florida in January 2016 and were attributed to travelers returning to the US, from infected areas in South America [22]. By February 2016, the World Health Organization (WHO) had declared the virus a global concern, leading to additional Facebook posts and official communications by the CDC and other government agencies [37]. In Quarter 3, data show that 61% and 39% of the posts in this timeframe received negative and positive feedback, respectively (Fig 3C). When assessing Quarter 4 (Fig 3D), data reflects observations in Quarter 2, with 62% and 38% of the posts in this timeframe receiving negative and positive feedback, respectively.

### Crisis and Emergency Risk Communication model

**Fig 4:**
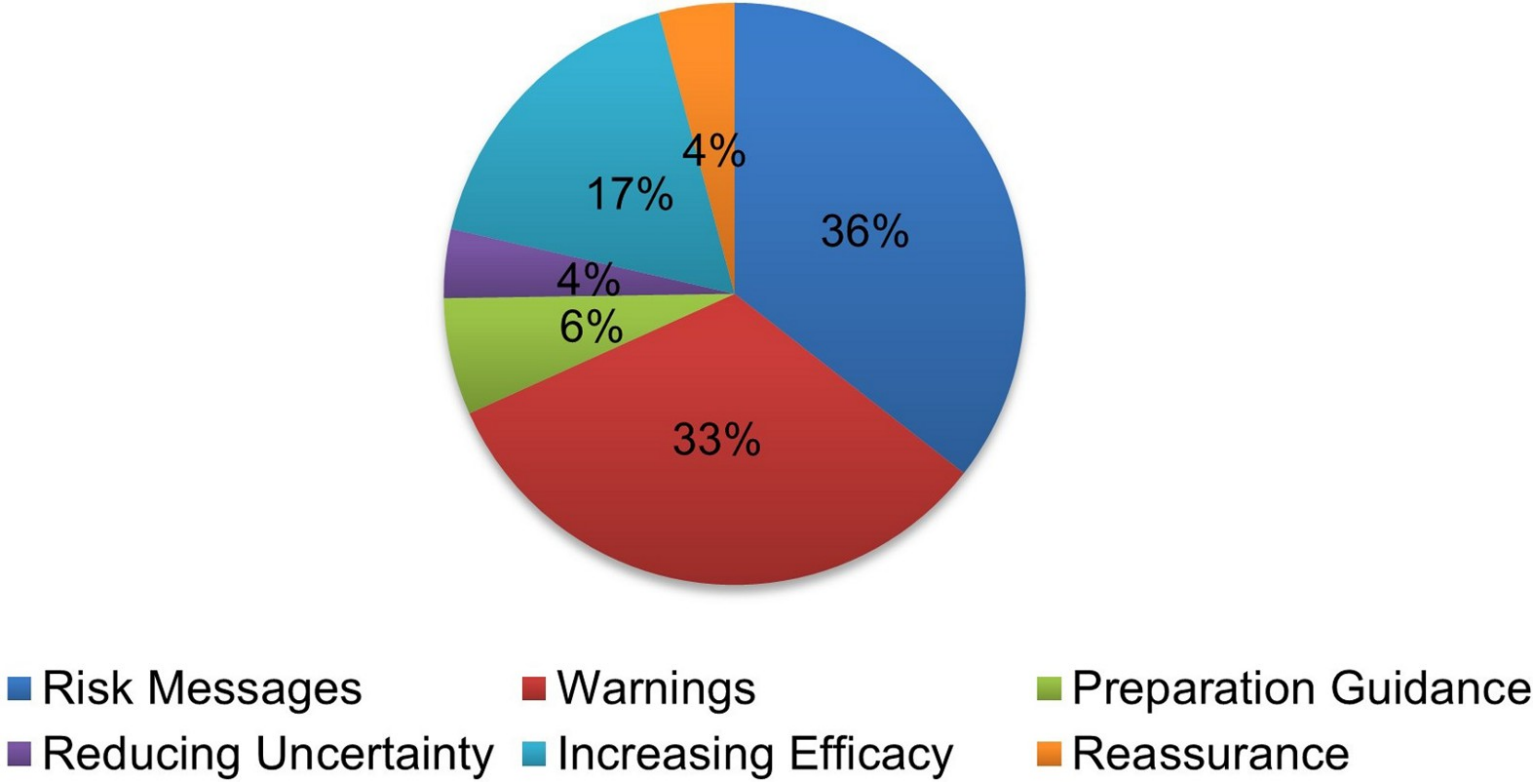
Percentage distribution of Crisis and Emergency Risk Communication (CERC) model themes (about Zika and dengue) found in federal health agency (i.e., CDC, FDA, NIAID, NIH) Facebook posts during 2016.

**Fig 5:**
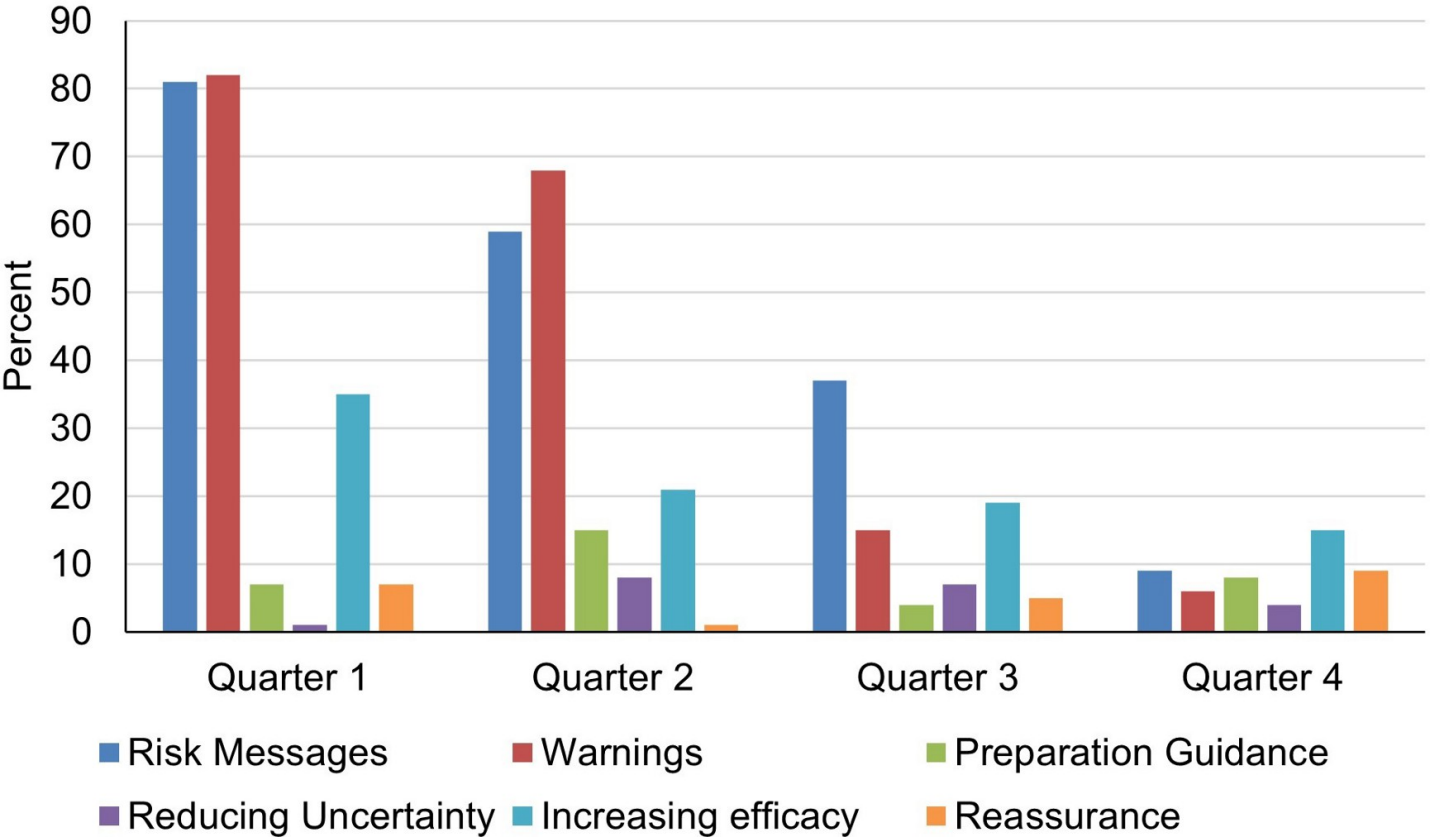
Quarterly Crisis and Emergency Risk Communication (CERC) Model themes (about Zika and dengue) found in government health agency (i.e., CDC, FDA, NIAID, NIH) Facebook posts during 2016.

Themes developed here from the CERC Model were adapted to the reviewed Facebook posts to further evaluate the effectiveness of the government agency posts (Figs 4 and 5) using the following criteria: 1) risk messages: general information about ZIKV/DENV, mosquitoes and virus transmission, and disease symptoms, n = 186 posts; 2) warnings: risk factors and dangers related to ZIKV/DENV, n = 171; 3) preparation guidance: health agencies responding to the disease and recommendations on how to respond, n = 34; 4) reducing uncertainty: human cases, local areas impacted, and sources of information, n = 20; 5) increasing efficacy: personal protection and disease prevention, n = 90; and 6) reassurance: comforting the public by specific government actions and recognizing the public’s efforts in preventing and fighting the disease, n = 22 posts.

## Discussion

Zika cases were first reported in Brazil in May 2015; however, Facebook posts communicated by US federal health agencies gained momentum in January 2016 (when data collection for the current study began). The reason for increased attention on ZIKV may have been due to the relationship between ZIKV infection and microcephaly in fetal development, an assertion first proposed by the Brazilian government in November 2015 and later supported [38, 39]. This was a direct concern to pregnant women, evidenced by social media posts collected for this study. The surge in social media posts in January 2016 can also be attributed to the spread of ZIKV which by then had already been detected in several countries outside Brazil [39]. The role of federal health agencies in providing timely and accurate information was evident in the amount of public engagement their posts received, especially at the height of the ZIKV outbreak. Posts likely aimed to address public fear of Zika (and dengue), especially in US residents and people traveling to areas impacted by the diseases (i.e., increase in posts about the diseases reflects public demand for information). An analysis on Facebook usage in Singapore during the 2016 Zika outbrak showed that public engagement increased in relation to outreach by respective health agencies [40].

Analyses provided insights into public perception of public health information published in Facebook. It is unlikely that everyone that viewed the posts also commented on the posts and this should be considered in interpretation of analyses. Most posts from the studied agencies occurred in Quarter 3 (July-September 2016) (n = 94 posts). In Quarter 3, 61% of government health agency posts received negative feedback and 39% received positive feedback, suggesting similar sentiment as Quarters 2 and 4. There was a noticeable difference in Facebook public feedback, considering the increasing negative sentiment from Quarter 1 (57%) to Quarter 4 (62%). While the increase of negative sentiment over time reflected a negative response to government social media posts as the pandemic progressed, the reasons for this are unclear and should be studied further. Public opinions (reflected in number of responses to federal health agency posts) seemed to plateau between Quarters 2 and 4, perhaps signaling settling public opinions. It should be noted that word clouds show the frequency of each commonly used word, but are not an indication of the importance or significance of each word for each studied time period. This tool quantified the qualitative data, but lacks the ability to expand on qualitative interpretations of these data. A study examining microblogs on a popular Chinese social media platform (Sina-Weibo) showed that Zika-related posts were most common in September 2016 (Quarter 3) [41].

Here, public Facebook comments and reactions to federal health agency posts contained personal opinions/feelings rather than official health information responses from the health agencies themselves. In several instances, public users conveyed distrust of the information published in the studied posts. For example, comments and questions posed by Facebook users suggest that the CDC is not a credible organization–characterizing the agency as being complicit of the outbreak of disease itself (i.e., responsible for spreading ZIKV), and in turn involved with the pharmaceutical industry in unfounded conspiracies. Examples of public comments (i.e., “because they [CDC] have unleashed it in time to go with their new vaccine for it”; “fake disease to justify spraying toxic chemicals on the public”) in response to a public question (“Why is this [ZIKV] here now?”) listed on CDC’s Facebook page in May 2016. Studies show that social influence may play a role in shaping individuals’ responses to disease outbreaks, highlighting the weight that Facebook public comments and responses might have on other individuals engaging with a government post [41]. There were multiple codes in the Facebook posts related to mosquitoes and vector-borne disease symptoms, suggesting that at least some Facebook users understood the relationship between mosquitoes as vectors that cause diseases. However, there were also multiple codes related to questioning or undermining the validity of health information, implying that some public users did not possess a clear understanding of the diseases and the information provided by federal health agencies. Others have shown that misleading Facebook posts about Zika were more popular than informative posts [24]. The same study showed the most popular useful and informative Facebook post by the World Health Organization (i.e., press release) had 43,000 views and 964 shares. Alternatively, the most popular misleading post (i.e., video entitled “10 reasons why Zika virus fear is a fraudulent medical hoax”) had > 530,000 views and 19,600 shares [24].

In the current study, Facebook users sometimes mentioned the topic of mosquitoes to inquire about protection practices and common symptoms. Federal health agencies answered some of these inquiries and provided additional information and links; however, this type of official response from the health agencies was infrequent (i.e., approximately one health agency response for every 10 public comments). Taking time to address social media user comments, questions, concerns, and statements, especially erroneous and possibly false statements, could help health agencies manage the conversation, and the quality/legitimacy of information presented on their official pages. Federal and other health agencies should consider routine review of social media posts to address public concerns in real time [41] so accurate information could be provided in social media platforms during epidemics.

A proactive approach of countering false information by the Italian Ministry of Health’s Facebook page was studied [42]. In light of increased misinformation spreading within the ministry’s own Facebook page about the Covid-19 pandemic, the official page ramped up efforts to counter fake news, dedicating 7.1% of the institutional flow between January 30—April 3, 2020 –in what was found to be the height of engagement from Facebook users during that country’s Covid-19 health crisis [42]. The same study found that the Ministry had to adopt specific digital communication strategies to face the Covid-19 pandemic, thereby devoting efforts to reduce misinformation. This was accomplished by using data and visuals to improve the effectiveness and comprehension of health information shared in its page. By April 2020, the Italian government had launched a task force to promote collaboration with fact-checkers and to encourage the public’s input in signaling and identifying misinformation on social media. A study in China showed that younger people and college-educated people were most likely to consider posts from health agencies as reliable [41]. A transparent, strategic, and proactive use of social media by public health agencies is an effective way to increase public trust and reduce the impact of false narratives [42]. Notable aspects of the current and other [43] studies are that a shortage of replies by a health agency to public comments on Facebook pages were observed, suggesting that people’s legitimate questions, doubts, and comments remained largely unanswered, therefore undermining trust in health agencies. Increasing health official engagement with the public to fill this void, such as hiring staff or developing committees that specifically focus on social media platform messaging compliance and metrics would be an important method to ensuring a strategic and proactive use of social media by health agencies.

Information shared in a social media platform (e.g., infographics) should be as important as textual posts, i.e., engagement should occur from the health agency responding to public comments. Posts about public health, disease surveillance, and control should be less of an advertisement (i.e., single post) and more of a forum–with careful monitoring and engagement with users. Leaders from public health agencies should create science-based messaging to improve public health education and risk communication [20]. Government agencies in Singapore were informative and engaged in their posts during the ZIKV outbreak, providing updates on ZIKV cases and on their vector control efforts [44]. In contrast with our findings, the study found that public response to government health information posts conveyed elements of care (e.g., highlighting the risks to pregnant women, and “elements of community such as well-wishes to other individuals”). In relation to crisis/risk communication, government agencies’ effective use of social media can help inform the public while also reducing uncertainty and promoting assurance [36]. Types of interactions may vary between different types of social media outlets. Another study found that Twitter was dominated by news media posts during the Zika outbreak, hence government health agencies may consider collaborating with news media outlets to provide information to the public [44].

Adopting existing frameworks such as the CERC program is another method to ensure strategic and proactive use of social media by public health agencies. When considering the themes from the CERC program, we noticed most of the studied posts fell under the risk message and warning themes, compared to the least frequent themes of reassurance and reducing uncertainty. Some posts contained a mixture of themes, containing information that met the criteria for one or more themes. While risk communication is important, especially for emerging diseases, providing the public with ample information about human cases and areas impacted is vital to: 1) reduce uncertainty, 2) provide insight into contact tracing, and 3) inform the public on travel restrictions. Agencies can adopt and/or adjust themes, depending on sentiment, content, and/or inquiries in public commenter posts to provide balanced information in health education campaigns. If there is misinformation in public comments about risks, health agencies could counter that with science-based information on risk and reassure the public of the appropriate public health information.

Adopting this method as a guideline or measure for compliance could help in strengthening agencies’ credibility among the public. Here, posts meeting the reassurance theme criteria were lacking, a troubling finding considering that information associated with this theme lets the public know what the federal health agency is doing to address a public health emergency. This is a critical strategy to improve if an organization seeks to maintain relevancy and justify public investment, public engagement, and disease prevention efforts.

Government health agencies can make use of CERC phases when forming health education campaigns so participants better understand needs of the media, other health organizations, private and public organizations, and the public impacted by a disease. Although this study is limited to ZIKV and DENV, the process can also be used to assess public perception to pandemics (e.g., Covid-19) and official federal health agency communications. Monitoring social media during crises also gives insight into public interactions with each other [45].

Here, there were numerous critical posts towards the CDC; however, this federal health agency had the largest following of Facebook users among the health agencies studied during the period of study. This suggests the CDC was considered an authoritative information source during the ZIKV outbreak. This should motivate an organization like the CDC to continue sharing health information and improving health education about disease outbreaks by finding ways to better engage with the public through social media platforms. Public agencies should consider becoming more active on social media during crisis situations [45]. Strengthening strategies to convey accurate social media information about emerging diseases, as well as combating misinformation will be increasingly important, especially since media and information sharing sites continue to grow outside of traditional news platforms.

## Limitations and future studies

One of the limitations of this study is not measuring public feedback through traditional forms of study such as a survey. Thus, the current data on public feedback is limited to self-selected participants who reacted to the social media posts in the given time frame when data was collected. Some people may not be inclined to comment on a post, even if they view the post positively or negatively. Additionally, the same people may not comment on posts each quarter, thereby making it more difficult to attribute shifts in sentiment over time. The current study does not consider factors which compel people to engage in the posts. Other considerations to note include social media platform limitations (i.e., assessing Facebook and not other social media platforms). Data were limited to available features of Facebook at the time of the study (i.e., Facebook has since changed the way it allows user interaction with posts and data, such as the expansion of the ‘like’ button features and addition of emotion icons that can help infer feedback and sentiment about a post, in addition to the content gathered from written comments in posts). The change (occurred in May 2017) from ‘like’ to ‘reactions’ (captures a wider array of reaction types) may also be useful for better understanding sentiments in future research. The data analyzed here is approximately six years old (when Zika outbreaks were widespread). Hence, future studies could quantify Facebook and/or other social media comments (e.g., % from agency and % from public commenters) and reactions to health agency posts periodically (during years with outbreaks or non-outbreak years). This could give agencies information on the degree of effort and resources needed to manage their social media accounts. Studies should focus on misinformation trends (i.e., public’s reaction and reception of emerging health information) and quantifying agencies’ efforts in responding to posts and countering misinformation. Future studies could examine various contextual factors that may influence people to comment or not comment on social media posts. Future studies could also examine public feedback to the agencies’ responses to combatting misinformation on their social media platforms. This will shape future health communication campaigns via social media.

## Conclusion

Our study demonstrates that Facebook can be a social media platform for distributing health information dissemination during a public health issue. Primary recommendations for health agencies include: 1) Increase focus on social media messaging and timely responses to public posts in a forum format, 2) Improve risk communication and reassure the public by countering misinformation with science-based health information, and 3) Continue to improve strategies to convey important health information to the public on a variety of social media platforms. Our findings show the importance of strategic risk communication that may vary during each stage of a disease outbreak. Social media is a novel and (if properly utilized) potentially useful means for health agencies to communicate with the public.

## Supporting information

S1 Text Tables A-F. Table A. Initial list of key word codes created to analyze the posts and to discover their frequency and relevancy. Table B. Automated themes, number of files*, and number of references** identified. *The number of files is the number (out of 214 total) that contained that theme and subtheme (subordinate codes). **The number of references is the amount of times any theme came in the context of the files (i.e., imported Facebook data/posts). Table C. Examples of parent themes with subordinate codes, number of files*, and number of references** identified. *The number of files is the number (out of 214 total) that contained that theme and subtheme (subordinate codes). **The number of references is the amount of times any theme came in the context of the files (i.e., imported Facebook data/posts). Table D. Sample of themes and subthemes. Table E. Sentiment assessment based on the number of codes found by post. Table F. Sentiment Assessment of Facebook posts.
(DOCX)
