# Supplementary material for: Facebook and mosquito-borne disease outbreaks: An analysis of public responses to federal health agencies’ posts about dengue and Zika in 2016
Source: PLOS Glob Public Health. 2022 Sep 12;2(9):e0000977. doi: 10.1371/journal.pgph.0000977 (PMC10022203; doi:10.1371/journal.pgph.0000977)
Supplement: S1 Text — Tables A-F. Table A. Initial list of key word codes created to analyze the posts and to discover their frequency and relevancy. Table B. Automated themes, number of files*, and number of references** identified. *The number of files is the number (out of 214 total) that contained that theme and subtheme (subordinate codes). **The number of references is the amount of times any theme came in the context of the files (i.e., imported Facebook data/posts). Table C. Examples of parent themes with subordinate codes, number of files*, and number of references** identified. *The number of files is the number (out of 214 total) that contained that theme and subtheme (subordinate codes). **The number of references is the amount of times any theme came in the context of the files (i.e., imported Facebook data/posts). Table D. Sample of themes and subthemes. Table E. Sentiment assessment based on the number of codes found by post. Table F. Sentiment Assessment of Facebook posts. (DOCX) [file pgph.0000977.s001.docx]

**Supporting Information**

Table A. Initial list of key word codes created to analyze the posts and to discover their frequency and relevancy.

| Key Word | Files | References |
| --- | --- | --- |
| #zika | 1 | 1 |
| aedes aegypti | 8 | 12 |
| aedes albopictus | 6 | 8 |
| avoid | 8 | 8 |
| birth-related | 1 | 1 |
| bite | 2 | 2 |
| biters | 1 | 1 |
| bites | 2 | 2 |
| bug | 3 | 4 |
| bugs | 198 | 1462 |
| care | 1 | 1 |
| cases | 20 | 34 |
| CDC negative | 25 | 94 |
| chikungunya | 17 | 19 |
| chikungunya, | 1 | 1 |
| Confirmed Symptoms | 1 | 1 |
| conspiracy. | 4 | 6 |
| control | 23 | 36 |
| Cover | 198 | 1463 |
| created | 5 | 6 |
| cross | 2 | 2 |
| DEET | 2 | 2 |
| dengue | 20 | 33 |
| dengue, | 1 | 1 |
| diarrhea | 1 | 1 |
| disease | 49 | 114 |
| diseases | 1 | 1 |
| Educate | 1 | 1 |
| evidence | 4 | 4 |
| experts | 1 | 1 |
| fearmongering~ | 1 | 1 |
| fetus | 1 | 1 |
| Florida | 5 | 6 |
| government | 24 | 32 |
| Government negative | 20 | 52 |
| Hispanics | 1 | 1 |
| infected | 1 | 1 |
| infection | 31 | 110 |
| insect repellent | 2 | 2 |
| Insecticide | 1 | 1 |
| IR3535 | 1 | 1 |
| Join | 1 | 1 |
| join us | 1 | 1 |
| labs | 4 | 4 |
| Learn | 13 | 16 |
| locally acquired | 16 | 30 |
| Lyme disease | 25 | 77 |
| Malaria | 12 | 15 |
| Microcephaly | 1 | 1 |
| mosquito | 2 | 3 |
| mosquito (2) | 1 | 1 |
| mosquito-borne disease | 20 | 36 |
| mosquito-borne diseases | 1 | 1 |
| mosquitoes | 43 | 86 |
| mosquitos | 1 | 1 |
| mother | 1 | 1 |
| non-pregnant | 2 | 3 |
| oil of lemon eucalyptus | 1 | 1 |
| outbreak | 198 | 1462 |
| pandemic | 6 | 8 |
| para-menthane-diol | 1 | 1 |
| picaridin | 1 | 1 |
| pregnancy | 2 | 2 |
| premature | 1 | 1 |
| prevent | 1 | 1 |
| preventing | 1 | 1 |
| Protect yourself | 3 | 3 |
| Public health | 23 | 51 |
| public information | 2 | 2 |
| Puerto Rico | 6 | 6 |
| repellent | 1 | 1 |
| reported | 21 | 34 |
| research | 14 | 18 |
| serious | 22 | 37 |
| Skin | 1 | 1 |
| spraying | 23 | 33 |
| spread | 26 | 52 |
| spread viruses | 2 | 2 |
| spreads | 10 | 11 |
| Standing water | 1 | 1 |
| stop | 10 | 11 |
| strain | 5 | 6 |
| support | 21 | 29 |
| Surveillance | 1 | 1 |
| symptoms | 10 | 13 |
| tick | 1 | 1 |
| transmit | 16 | 37 |
| treat | 1 | 1 |
| vector | 0 | 0 |
| vectors | 9 | 10 |
| virus | 15 | 26 |
| viruses | 39 | 261 |
| Wear | 198 | 1462 |
| West Nile Virus | 17 | 50 |
| yellow fever | 14 | 22 |
| Zika | 53 | 366 |
| Zika virus | 22 | 34 |

Table B. Automated themes, number of files*, and number of references** identified. *The number of files is the number (out of 214 total) that contained that theme and subtheme (subordinate codes). **The number of references is the amount of times any theme came in the context of the files (i.e., imported Facebook data/posts).

| Name | Files | References |
| --- | --- | --- |
| active ingredients | 5 | 8 |
| aedes | 5 | 6 |
| aedes species | 19 | 19 |
| affected | 1 | 2 |
| antibody | 4 | 8 |
| areas | 1 | 2 |
| babies | 1 | 2 |
| birth | 8 | 12 |
| birth defects | 34 | 67 |
| bite | 2 | 4 |
| bites | 40 | 55 |
| blood | 7 | 12 |
| brain | 12 | 18 |
| brazilian doctors | 1 | 1 |
| bug spray | 3 | 5 |
| cells | 3 | 5 |
| compounds | 1 | 2 |
| control | 3 | 6 |
| current travel notices | 1 | 2 |
| defected | 1 | 2 |
| delivery | 1 | 2 |
| dengue | 4 | 15 |
| dengue vaccine | 2 | 4 |
| dengue virus | 2 | 5 |
| developing fetuses | 2 | 2 |
| development | 4 | 4 |
| disease | 27 | 48 |
| diseases | 8 | 12 |
| donor blood | 1 | 2 |
| drug | 8 | 18 |
| early-stage study | 1 | 2 |
| effect | 11 | 14 |
| effective | 4 | 6 |
| effects | 18 | 23 |
| epidemic | 1 | 2 |
| evidence | 14 | 22 |
| experimental | 4 | 4 |
| experimental vaccine | 2 | 3 |
| food | 4 | 6 |
| funding | 2 | 4 |
| government | 11 | 12 |
| groups | 2 | 5 |
| guidelines | 1 | 2 |
| heads | 2 | 2 |
| health | 42 | 63 |
| health risks | 2 | 2 |
| health threat | 1 | 2 |
| human | 9 | 12 |
| immune | 3 | 4 |
| infected | 1 | 2 |
| infection | 40 | 67 |
| information | 10 | 15 |
| insect | 7 | 12 |
| insect repellents | 10 | 31 |
| label | 7 | 8 |
| lemon | 4 | 7 |
| mice | 2 | 2 |
| money | 2 | 2 |
| mosq* | 6 | 6 |
| mosquito | 78 | 144 |
| mosquito bites | 36 | 45 |
| mosquitoes | 3 | 5 |
| notices | 1 | 2 |
| oil | 1 | 4 |
| oils | 7 | 8 |
| outbreak | 2 | 2 |
| pain | 15 | 20 |
| people | 18 | 20 |
| placebo | 1 | 4 |
| potential cause | 1 | 1 |
| pregnant | 42 | 61 |
| pregnant women | 28 | 36 |
| pretense | 1 | 2 |
| products | 16 | 24 |
| protein | 3 | 5 |
| related flavivirus | 1 | 2 |
| repellant | 6 | 9 |
| repellants | 3 | 4 |
| research | 6 | 6 |
| researchers | 4 | 4 |
| respiratory tract | 1 | 1 |
| response | 3 | 4 |
| right | 1 | 2 |
| risk | 1 | 2 |
| safety | 7 | 10 |
| science | 1 | 2 |
| scientific process | 1 | 2 |
| sexual transmission | 1 | 4 |
| share | 5 | 5 |
| specimens | 1 | 2 |
| spraying | 22 | 34 |
| stinger | 1 | 2 |
| study | 12 | 14 |
| supply | 7 | 7 |
| sweat odor | 1 | 2 |
| system | 9 | 10 |
| test | 3 | 5 |
| testing | 1 | 2 |
| tests | 17 | 25 |
| tobacco | 7 | 7 |
| transmission | 26 | 36 |
| travel | 21 | 30 |
| trials | 1 | 2 |
| use | 10 | 12 |
| vaccine | 44 | 89 |
| vaccine candidate | 2 | 2 |
| virus | 59 | 100 |
| viruses | 9 | 13 |
| water | 1 | 2 |
| women | 27 | 36 |
| yellow fever | 7 | 13 |
| zika | 29 | 38 |
| zika virus | 6 | 6 |

Table C. Examples of parent themes with subordinate codes, number of files*, and number of references** identified. *The number of files is the number (out of 214 total) that contained that theme and subtheme (subordinate codes). **The number of references is the amount of times any theme came in the context of the files (i.e., imported Facebook data/posts).

| Name | Files | References |
| --- | --- | --- |
| Infection | 40 | 67 |
| active infection | 1 | 2 |
| acute infection | 1 | 1 |
| asymptomatic infections | 1 | 1 |
| certain infections | 1 | 1 |
| chronic infection | 2 | 2 |
| congenital infection | 1 | 1 |
| determining infection | 1 | 1 |
| elizabethkingia infection | 1 | 1 |
| future infections | 2 | 2 |
| hospital-acquired infections | 1 | 1 |
| infect others | 1 | 1 |
| infected animals | 1 | 1 |
| infected man | 5 | 5 |
| infected mosquito | 2 | 2 |
| infected mosquitoes | 1 | 1 |
| infected partner | 1 | 1 |
| infected person | 5 | 6 |
| infected women | 1 | 1 |
| infection control advice | 1 | 1 |
| infection projections | 1 | 1 |
| infection rates | 1 | 1 |
| intrauterine infection | 1 | 1 |
| lung infection | 1 | 1 |
| malaria-like infection | 1 | 1 |
| maternal infection | 1 | 1 |
| mild infection | 1 | 1 |
| norovirus infections | 1 | 1 |
| paralytic infection | 1 | 1 |
| persistent infection | 1 | 1 |
| possible infections | 1 | 1 |
| real infection | 1 | 1 |
| rubella infection | 1 | 1 |
| secondary infect | 1 | 1 |
| symptomatic cytomegalovirus Infections | 1 | 1 |
| tick infections | 1 | 1 |
| tick-borne infection | 1 | 1 |
| transfusion-transmitted infection | 1 | 1 |
| transmitted infection | 1 | 1 |
| trimester infections | 1 | 1 |
| uncomplicated infections | 1 | 2 |
| viral infections | 1 | 1 |
| virus infection | 8 | 9 |
| zika infections | 3 | 3 |
| Information | 10 | 15 |
| available information | 1 | 1 |
| complete information | 1 | 1 |
| contact information | 3 | 3 |
| forward information | 1 | 1 |
| genetic information | 1 | 1 |
| important health information | 1 | 1 |
| important information | 1 | 1 |
| including safety information | 1 | 1 |
| key information | 1 | 1 |
| releasable information | 1 | 1 |
| travel information | 1 | 1 |

Table D. Sample of themes and subthemes

| Theme | Subtheme |
| --- | --- |
| Mosquito | A.1 mosquito bites  A.2 aedes species mosquito  A.3 controlling mosquitoes  A.4 mosquito spray  A.5 mosquito population  A.6 Carrying mosquitoes  A.7 infected mosquito  A.8 standard mosquito control  A.9 mosquito larva  A.10 avoiding mosquito bites  A. 11 adult mosquitoes  A.12 albopictus mosquitoes  A.13 mosquito control measures  A.14 mosquito season  A.15 aegypti mosquitoes  A.16 mosquito control efforts  A.17 mosquito repellant  A.18 good mosquito repellant  A.19 zika mosquitoes  A.20 mosquito netting |
| Virus | B.1 zika virus  B.2 virus infection  B.3 virus transmission  B.4 chikungunya viruses  B.5 virus outbreak  B.6 virus exposure  B.7 mosquito-borne virus  B.8 virus disease  B.9 dengue virus  B.10 flu virus  B.11 spread viruses  B.12 yellow fever viruses  B.13 super zika virus  B.14 infectious virus  B.15 virus threat  B.16 zika virus outbreak  B.17 virus vaccines  B.18 virus updates  B.19 vector-borne virus  B.20 money making virus |

Table E. Sentiment assessment based on the number of codes found by post.

|  | A : Very negative | B : Moderately negative | C : Moderately positive | D : Very positive |
| --- | --- | --- | --- | --- |
| 1 : Files\\CDC - Dengue - 07.07.16 | 0 | 124 | 86 | 0 |
| 2 : Files\\CDC - Dengue - 07.07.16b | 0 | 0 | 0 | 0 |
| 3 : Files\\CDC - Dengue - 07.13.16 | 0 | 0 | 25 | 0 |
| 4 : Files\\CDC - Dengue - 08.02.16 | 16 | 9 | 48 | 60 |
| 5 : Files\\CDC - Dengue - 08.02.16b | 0 | 0 | 0 | 0 |
| 6 : Files\\CDC - Dengue - 08.15.16 | 456 | 488 | 259 | 182 |
| 7 : Files\\CDC - Dengue - 08.24.16 | 150 | 240 | 100 | 79 |
| 8 : Files\\CDC - Dengue - 09.26.16 | 92 | 67 | 20 | 0 |
| 9 : Files\\CDC - Dengue - 12.14.16 | 460 | 124 | 209 | 33 |
| 10 : Files\\CDC - Zika - 01.25.16 | 356 | 204 | 141 | 144 |
| 11 : Files\\CDC - Zika - 01.28.16 | 483 | 566 | 318 | 86 |
| 12 : Files\\CDC - Zika - 02.05.16 | 293 | 9 | 65 | 183 |
| 13 : Files\\CDC - Zika - 02.08.16 | 263 | 231 | 116 | 6 |
| 14 : Files\\CDC - Zika - 02.09.16 | 73 | 128 | 87 | 82 |
| 15 : Files\\CDC - Zika - 02.10.16 | 14 | 22 | 74 | 117 |
| 16 : Files\\CDC - Zika - 02.11.16 | 235 | 282 | 242 | 188 |
| 17 : Files\\CDC - Zika - 02.16.16 | 147 | 190 | 66 | 0 |
| 18 : Files\\CDC - Zika - 02.19.16 | 0 | 0 | 0 | 0 |
| 19 : Files\\CDC - Zika - 02.19.16 (2) | 0 | 65 | 0 | 0 |
| 20 : Files\\CDC - Zika - 02.21.16 | 27 | 77 | 61 | 0 |
| 21 : Files\\CDC - Zika - 02.22.16 | 152 | 123 | 77 | 63 |
| 22 : Files\\CDC - Zika - 02.23.16 | 249 | 277 | 108 | 7 |
| 23 : Files\\CDC - Zika - 03.04.16 | 490 | 673 | 556 | 177 |
| 24 : Files\\CDC - Zika - 03.06.16 | 129 | 133 | 83 | 39 |
| 25 : Files\\CDC - Zika - 03.06.16 (2) | 69 | 4 | 63 | 0 |
| 26 : Files\\CDC - Zika - 03.12.16 | 0 | 15 | 0 | 12 |
| 27 : Files\\CDC - Zika - 03.14.16 | 0 | 15 | 0 | 0 |
| 28 : Files\\CDC - Zika - 03.24.16 | 27 | 168 | 12 | 14 |
| 29 : Files\\CDC - Zika - 03.25.16 | 117 | 0 | 83 | 12 |
| 30 : Files\\CDC - Zika - 04.01.16 | 452 | 486 | 529 | 185 |
| 31 : Files\\CDC - Zika - 04.08.16 | 229 | 46 | 25 | 93 |
| 32 : Files\\CDC - Zika - 04.11.16 | 781 | 1189 | 287 | 85 |
| 33 : Files\\CDC - Zika - 04.13.16 | 598 | 750 | 538 | 140 |
| 34 : Files\\CDC - Zika - 04.19.16 | 46 | 225 | 0 | 43 |
| 35 : Files\\CDC - Zika - 04.20.16 | 46 | 124 | 84 | 43 |
| 36 : Files\\CDC - Zika - 04.27.16 | 0 | 0 | 41 | 0 |
| 37 : Files\\CDC - Zika - 05.13.15 | 151 | 62 | 89 | 0 |
| 38 : Files\\CDC - Zika - 05.15.16 | 256 | 191 | 57 | 50 |
| 39 : Files\\CDC - Zika - 05.20.16 | 0 | 0 | 0 | 0 |
| 40 : Files\\CDC - Zika - 05.22.15 | 25 | 0 | 0 | 17 |
| 41 : Files\\CDC - Zika - 05.25.16 | 0 | 0 | 0 | 0 |
| 42 : Files\\CDC - Zika - 05.31.16 | 0 | 0 | 0 | 58 |
| 43 : Files\\CDC - Zika - 06.03.16 | 0 | 62 | 62 | 5 |
| 44 : Files\\CDC - Zika - 06.07.16 | 887 | 1761 | 320 | 647 |
| 45 : Files\\CDC - Zika - 06.08.16 | 180 | 293 | 278 | 101 |
| 46 : Files\\CDC - Zika - 06.08.16 (2) | 205 | 197 | 47 | 109 |
| 47 : Files\\CDC - Zika - 06.13.16 | 186 | 106 | 61 | 107 |
| 48 : Files\\CDC - Zika - 07.04.16 | 86 | 0 | 0 | 55 |
| 49 : Files\\CDC - Zika - 07.06.16 | 95 | 116 | 40 | 13 |
| 50 : Files\\CDC - Zika - 07.11.16 | 0 | 24 | 24 | 0 |
| 51 : Files\\CDC - Zika - 07.18.16 | 12 | 0 | 0 | 0 |
| 52 : Files\\CDC - Zika - 07.25.16 | 35 | 53 | 56 | 0 |
| 53 : Files\\CDC - Zika - 07.27.16 | 107 | 25 | 55 | 1 |
| 54 : Files\\CDC - Zika - 07.29.16 | 331 | 166 | 137 | 139 |
| 55 : Files\\CDC - Zika - 07.30.16 | 95 | 33 | 107 | 28 |
| 56 : Files\\CDC - Zika - 08.01.16 | 369 | 262 | 238 | 88 |
| 57 : Files\\CDC - Zika - 08.03.16 | 0 | 39 | 26 | 0 |
| 58 : Files\\CDC - Zika - 08.13.16 | 261 | 144 | 80 | 45 |
| 59 : Files\\CDC - Zika - 08.18.16 | 91 | 78 | 36 | 0 |
| 60 : Files\\CDC - Zika - 08.18.16 (2) | 286 | 276 | 214 | 70 |
| 61 : Files\\CDC - Zika - 08.19.16 | 59 | 110 | 25 | 0 |
| 62 : Files\\CDC - Zika - 08.23.16 | 183 | 208 | 122 | 73 |
| 63 : Files\\CDC - Zika - 08.25.16 | 0 | 0 | 0 | 18 |
| 64 : Files\\CDC - Zika - 08.26.16 | 0 | 0 | 0 | 0 |
| 65 : Files\\CDC - Zika - 08.27.16 | 173 | 88 | 127 | 23 |
| 66 : Files\\CDC - Zika - 08.30.16 | 119 | 52 | 0 | 0 |
| 67 : Files\\CDC - Zika - 08.31.16 | 560 | 820 | 230 | 271 |
| 68 : Files\\CDC - Zika - 09.09.16 | 490 | 277 | 154 | 336 |
| 69 : Files\\CDC - Zika - 09.11.16 | 215 | 72 | 0 | 25 |
| 70 : Files\\CDC - Zika - 09.14.16 | 13 | 0 | 0 | 0 |
| 71 : Files\\CDC - Zika - 09.15.16 | 186 | 0 | 28 | 0 |
| 72 : Files\\CDC - Zika - 09.19.16 | 103 | 231 | 194 | 0 |
| 73 : Files\\CDC - Zika - 09.22.16 | 0 | 18 | 23 | 0 |
| 74 : Files\\CDC - Zika - 09.22.16 (2) | 74 | 125 | 74 | 0 |
| 75 : Files\\CDC - Zika - 09.23.16 | 10 | 253 | 157 | 23 |
| 76 : Files\\CDC - Zika - 09.24.16 | 0 | 29 | 0 | 25 |
| 77 : Files\\CDC - Zika - 09.25.16 | 419 | 213 | 187 | 65 |
| 78 : Files\\CDC - Zika - 09.25.16 (2) | 109 | 57 | 22 | 13 |
| 79 : Files\\CDC - Zika - 09.26.16 | 113 | 65 | 54 | 0 |
| 80 : Files\\CDC - Zika - 10.11.16 | 130 | 65 | 178 | 61 |
| 81 : Files\\CDC - Zika - 10.13.16 | 82 | 56 | 50 | 41 |
| 82 : Files\\CDC - Zika - 10.18.16 | 390 | 609 | 153 | 169 |
| 83 : Files\\CDC - Zika - 10.19.16 | 113 | 57 | 0 | 50 |
| 84 : Files\\CDC - Zika - 11.12.16 | 50 | 102 | 46 | 20 |
| 85 : Files\\CDC - Zika - 11.28.16 | 845 | 1079 | 597 | 326 |
| 86 : Files\\CDC - Zika - 12.15.16 | 202 | 170 | 20 | 124 |
| 87 : Files\\CDC - Zika - 12.19.16 | 298 | 923 | 303 | 95 |
| 88 : Files\\CDC - Zika - 12.22.16 | 269 | 435 | 177 | 34 |
| 89 : Files\\CDC - Zika - 12.29.16 | 323 | 913 | 329 | 132 |
| 90 : Files\\CDC - Zika - 12.29.16 (2) | 366 | 401 | 325 | 67 |
| 91 : Files\\FDA - Dengue - 08.17.16 | 386 | 226 | 248 | 41 |
| 92 : Files\\FDA - Dengue - 12.16.16 | 0 | 0 | 0 | 0 |
| 93 : Files\\FDA- Zika - 02.16.16 | 0 | 0 | 184 | 11 |
| 94 : Files\\FDA- Zika - 02.17.16 | 0 | 0 | 0 | 0 |
| 95 : Files\\FDA- Zika - 02.23.16 | 0 | 0 | 0 | 0 |
| 96 : Files\\FDA- Zika - 02.26.16 | 44 | 0 | 191 | 4 |
| 97 : Files\\FDA- Zika - 02.26.16 (2) | 81 | 75 | 38 | 0 |
| 98 : Files\\FDA- Zika - 03.02.16 | 0 | 0 | 57 | 0 |
| 99 : Files\\FDA- Zika - 03.30.16 | 38 | 73 | 36 | 84 |
| 100 : Files\\FDA- Zika - 04.04.16 | 37 | 36 | 49 | 0 |
| 101 : Files\\FDA- Zika - 04.06.16 | 27 | 0 | 0 | 0 |
| 102 : Files\\FDA- Zika - 04.29.16 | 60 | 127 | 0 | 59 |
| 103 : Files\\FDA- Zika - 05.16.16 | 0 | 0 | 29 | 0 |
| 104 : Files\\FDA- Zika - 06.13.16 | 126 | 0 | 41 | 126 |
| 105 : Files\\FDA- Zika - 06.17.16 | 0 | 0 | 19 | 0 |
| 106 : Files\\FDA- Zika - 06.21.16 | 0 | 0 | 0 | 0 |
| 107 : Files\\FDA- Zika - 06.21.16 (2) | 23 | 0 | 0 | 0 |
| 108 : Files\\FDA- Zika - 06.22.16 | 0 | 26 | 0 | 0 |
| 109 : Files\\FDA- Zika - 06.24.16 | 69 | 81 | 34 | 69 |
| 110 : Files\\FDA- Zika - 06.24.16 (2) | 0 | 10 | 0 | 0 |
| 111 : Files\\FDA- Zika - 06.27.16 | 25 | 70 | 39 | 0 |
| 112 : Files\\FDA- Zika - 06.29.16 | 25 | 0 | 0 | 0 |
| 113 : Files\\FDA- Zika - 07.05.16 | 0 | 0 | 0 | 0 |
| 114 : Files\\FDA- Zika - 07.12.16 | 0 | 93 | 0 | 0 |
| 115 : Files\\FDA- Zika - 07.12.16 picture | 0 | 0 | 0 | 0 |
| 116 : Files\\FDA- Zika - 07.20.16 | 0 | 0 | 13 | 54 |
| 117 : Files\\FDA- Zika - 07.21.16 | 165 | 50 | 54 | 39 |
| 118 : Files\\FDA- Zika - 07.22.16 | 114 | 0 | 0 | 0 |
| 119 : Files\\FDA- Zika - 07.26.16 | 0 | 22 | 0 | 0 |
| 120 : Files\\FDA- Zika - 07.28.16 | 0 | 0 | 17 | 0 |
| 121 : Files\\FDA- Zika - 07.29.16 | 26 | 0 | 0 | 50 |
| 122 : Files\\FDA- Zika - 07.29.16 (2) | 0 | 40 | 0 | 0 |
| 123 : Files\\FDA- Zika - 08.02.16 | 31 | 0 | 30 | 0 |
| 124 : Files\\FDA- Zika - 08.08.16 | 60 | 37 | 26 | 172 |
| 125 : Files\\FDA- Zika - 08.17.16 | 144 | 28 | 31 | 16 |
| 126 : Files\\FDA- Zika - 08.17.16 (2) | 417 | 225 | 247 | 41 |
| 127 : Files\\FDA- Zika - 08.18.16 | 0 | 53 | 28 | 0 |
| 128 : Files\\FDA- Zika - 08.19.16 | 1 | 67 | 0 | 0 |
| 129 : Files\\FDA- Zika - 08.19.16 (2) | 79 | 25 | 77 | 7 |
| 130 : Files\\FDA- Zika - 08.23.16 | 27 | 28 | 44 | 85 |
| 131 : Files\\FDA- Zika - 08.24.16 | 0 | 0 | 0 | 0 |
| 132 : Files\\FDA- Zika - 08.25.16 | 0 | 5 | 0 | 15 |
| 133 : Files\\FDA- Zika - 08.26.16 | 43 | 0 | 0 | 0 |
| 134 : Files\\FDA- Zika - 08.26.16 (2) | 95 | 72 | 16 | 0 |
| 135 : Files\\FDA- Zika - 08.26.16 (3) | 14 | 0 | 0 | 0 |
| 136 : Files\\FDA- Zika - 08.30.16 | 0 | 0 | 19 | 0 |
| 137 : Files\\FDA- Zika - 08.31.16 | 0 | 35 | 66 | 10 |
| 138 : Files\\FDA- Zika - 08.31.16 (2) | 26 | 89 | 26 | 12 |
| 139 : Files\\FDA- Zika - 09.02.16 | 95 | 58 | 48 | 0 |
| 140 : Files\\FDA- Zika - 09.03.16 | 147 | 187 | 42 | 0 |
| 141 : Files\\FDA- Zika - 09.07.16 | 0 | 0 | 0 | 0 |
| 142 : Files\\FDA- Zika - 09.09.16 | 0 | 31 | 0 | 0 |
| 143 : Files\\FDA- Zika - 09.12.16 | 0 | 103 | 210 | 13 |
| 144 : Files\\FDA- Zika - 09.14.16 | 25 | 0 | 6 | 0 |
| 145 : Files\\FDA- Zika - 09.16.16 | 0 | 0 | 0 | 0 |
| 146 : Files\\FDA- Zika - 09.17.16 | 27 | 96 | 96 | 47 |
| 147 : Files\\FDA- Zika - 09.22.16 | 47 | 0 | 41 | 85 |
| 148 : Files\\FDA- Zika - 09.23.16 | 0 | 48 | 0 | 0 |
| 149 : Files\\FDA- Zika - 09.26.16 | 90 | 21 | 12 | 0 |
| 150 : Files\\FDA- Zika - 10.04.16 | 72 | 151 | 113 | 45 |
| 151 : Files\\FDA- Zika - 10.07.16 | 100 | 69 | 155 | 0 |
| 152 : Files\\FDA- Zika - 10.14.16 | 0 | 22 | 14 | 0 |
| 153 : Files\\FDA- Zika - 10.17.16 | 0 | 28 | 0 | 0 |
| 154 : Files\\FDA- Zika - 11.01.16 | 114 | 177 | 266 | 42 |
| 155 : Files\\FDA- Zika - 11.04.16 | 0 | 0 | 0 | 0 |
| 156 : Files\\FDA- Zika - 11.08.16 | 0 | 0 | 0 | 0 |
| 157 : Files\\FDA- Zika - 11.17.16 | 0 | 0 | 0 | 0 |
| 158 : Files\\FDA- Zika - 11.22.16 | 59 | 84 | 85 | 62 |
| 159 : Files\\FDA- Zika - 11.25.16 | 101 | 33 | 15 | 76 |
| 160 : Files\\FDA- Zika - 11.29.16 | 0 | 0 | 0 | 0 |
| 161 : Files\\FDA- Zika - 11.30.16 | 0 | 56 | 48 | 0 |
| 162 : Files\\FDA- Zika - 12.02.16 | 67 | 44 | 16 | 0 |
| 163 : Files\\FDA- Zika - 12.06.16 | 0 | 58 | 58 | 0 |
| 164 : Files\\FDA- Zika - 12.08.16 | 36 | 0 | 0 | 18 |
| 165 : Files\\FDA- Zika - 12.09.16 | 0 | 0 | 0 | 5 |
| 166 : Files\\FDA- Zika - 12.13.16 | 0 | 15 | 0 | 0 |
| 167 : Files\\FDA- Zika - 12.16.16 | 0 | 0 | 77 | 0 |
| 168 : Files\\FDA- Zika - 12.20.16 | 83 | 74 | 18 | 11 |
| 169 : Files\\FDA- Zika - 12.26.16 | 0 | 16 | 0 | 29 |
| 170 : Files\\FDA- Zika - 12.29.16 | 0 | 0 | 0 | 0 |
| 171 : Files\\FDA- Zika - 12.30.16 | 0 | 0 | 0 | 0 |
| 172 : Files\\NIAID - Dengue - 01.14.16 | 0 | 14 | 67 | 2 |
| 173 : Files\\NIAID - Dengue - 03.16.16 | 57 | 43 | 90 | 0 |
| 174 : Files\\NIAID - Dengue - 08.16.16 | 0 | 18 | 0 | 0 |
| 175 : Files\\NIAID - Dengue - 08.17.16 | 19 | 11 | 0 | 58 |
| 176 : Files\\NIAID - Zika - 02.10.16 | 0 | 0 | 0 | 51 |
| 177 : Files\\NIAID - Zika - 03.31.16 | 13 | 0 | 15 | 0 |
| 178 : Files\\NIAID - Zika - 05.11.16 | 49 | 0 | 26 | 0 |
| 179 : Files\\NIAID - Zika - 05.17.16 | 0 | 0 | 0 | 0 |
| 180 : Files\\NIAID - Zika - 05.19.16 | 0 | 39 | 77 | 0 |
| 181 : Files\\NIAID - Zika - 06.21.16 | 0 | 62 | 0 | 0 |
| 182 : Files\\NIAID - Zika - 06.24.16 | 0 | 10 | 0 | 0 |
| 183 : Files\\NIAID - Zika - 06.28.16 | 0 | 0 | 0 | 0 |
| 184 : Files\\NIAID - Zika - 07.27.16 | 0 | 0 | 56 | 0 |
| 185 : Files\\NIAID - Zika - 08.04.16 | 0 | 0 | 66 | 0 |
| 186 : Files\\NIAID - Zika - 08.15.16 | 0 | 27 | 0 | 0 |
| 187 : Files\\NIAID - Zika - 08.15.16 (2) | 0 | 0 | 19 | 38 |
| 188 : Files\\NIAID - Zika - 08.15.16 (3) | 0 | 0 | 25 | 0 |
| 189 : Files\\NIAID - Zika - 08.15.16 (4) | 0 | 0 | 0 | 30 |
| 190 : Files\\NIAID - Zika - 08.16.16 | 141 | 172 | 158 | 124 |
| 191 : Files\\NIAID - Zika - 08.16.16 (2) | 0 | 18 | 0 | 0 |
| 192 : Files\\NIAID - Zika - 08.17.16 | 19 | 11 | 0 | 58 |
| 193 : Files\\NIAID - Zika - 09.22.16 | 0 | 0 | 34 | 0 |
| 194 : Files\\NIAID - Zika - 09.28.16 | 0 | 0 | 0 | 0 |
| 195 : Files\\NIAID - Zika - 11.04.16 | 117 | 0 | 0 | 117 |
| 196 : Files\\NIAID - Zika - 11.07.16 | 0 | 29 | 0 | 0 |
| 197 : Files\\NIAID - Zika - 11.07.16 (2) | 0 | 32 | 32 | 0 |
| 198 : Files\\NIAID - Zika - 11.17.16 | 31 | 46 | 0 | 31 |
| 199 : Files\\NIAID - Zika - 12.29.16 | 0 | 0 | 0 | 0 |
| 200 : Files\\NIH - Dengue - 03.16.16 | 34 | 51 | 39 | 111 |
| 201 : Files\\NIH - Zika - 01.25.16 | 64 | 68 | 0 | 0 |
| 202 : Files\\NIH - Zika - 03.15.16 | 0 | 38 | 0 | 0 |
| 203 : Files\\NIH - Zika - 04.23.16 | 0 | 0 | 0 | 0 |
| 204 : Files\\NIH - Zika - 05.05.16 | 65 | 71 | 145 | 45 |
| 205 : Files\\NIH - Zika - 05.19.16 | 46 | 0 | 0 | 46 |
| 206 : Files\\NIH - Zika - 06.10.16 | 0 | 0 | 0 | 0 |
| 207 : Files\\NIH - Zika - 06.28.16 | 0 | 0 | 90 | 0 |
| 208 : Files\\NIH - Zika - 06.28.16 (2) | 0 | 67 | 0 | 0 |
| 209 : Files\\NIH - Zika - 07.05.16 | 0 | 48 | 0 | 0 |
| 210 : Files\\NIH - Zika - 08.16.16 | 58 | 50 | 119 | 46 |
| 211 : Files\\NIH - Zika - 09.07.16 | 66 | 76 | 0 | 0 |
| 212 : Files\\NIH - Zika - 10.14.16 | 30 | 42 | 0 | 0 |
| 213 : Files\\NIH - Zika - 11.08.16 | 61 | 0 | 54 | 0 |
| 214 : Files\\NIH - Zika - 11.26.16 | 0 | 0 | 0 | 59 |

Table F. Sentiment Assessment of Facebook posts.

| Post | Very negative | Moderately negative | Moderately positive | Very positive | Positive | Negative |
| --- | --- | --- | --- | --- | --- | --- |
| 1 : Files\\CDC - Dengue - 07.07.16 | 0% | 59.05% | 40.95% | 0% | 40.95% | 59.05% |
| 2 : Files\\CDC - Dengue - 07.07.16b | 0% | 0% | 0% | 0% | 0.00% | 0.00% |
| 3 : Files\\CDC - Dengue - 07.13.16 | 0% | 0% | 100% | 0% | 100.00% | 0.00% |
| 4 : Files\\CDC - Dengue - 08.02.16 | 12.03% | 6.77% | 36.09% | 45.11% | 81.20% | 18.80% |
| 5 : Files\\CDC - Dengue - 08.02.16b | 0% | 0% | 0% | 0% | 0.00% | 0.00% |
| 6 : Files\\CDC - Dengue - 08.15.16 | 32.92% | 35.23% | 18.7% | 13.14% | 31.84% | 68.15% |
| 7 : Files\\CDC - Dengue - 08.24.16 | 26.36% | 42.18% | 17.57% | 13.88% | 31.45% | 68.54% |
| 8 : Files\\CDC - Dengue - 09.26.16 | 51.4% | 37.43% | 11.17% | 0% | 11.17% | 88.83% |
| 9 : Files\\CDC - Dengue - 12.14.16 | 55.69% | 15.01% | 25.3% | 4% | 29.30% | 70.70% |
| 10 : Files\\CDC - Zika - 01.25.16 | 42.13% | 24.14% | 16.69% | 17.04% | 33.73% | 66.27% |
| 11 : Files\\CDC - Zika - 01.28.16 | 33.24% | 38.95% | 21.89% | 5.92% | 27.81% | 72.19% |
| 12 : Files\\CDC - Zika - 02.05.16 | 53.27% | 1.64% | 11.82% | 33.27% | 45.09% | 54.91% |
| 13 : Files\\CDC - Zika - 02.08.16 | 42.69% | 37.5% | 18.83% | 0.97% | 19.80% | 80.19% |
| 14 : Files\\CDC - Zika - 02.09.16 | 19.73% | 34.59% | 23.51% | 22.16% | 45.67% | 54.32% |
| 15 : Files\\CDC - Zika - 02.10.16 | 6.17% | 9.69% | 32.6% | 51.54% | 84.14% | 15.86% |
| 16 : Files\\CDC - Zika - 02.11.16 | 24.82% | 29.78% | 25.55% | 19.85% | 45.40% | 54.60% |
| 17 : Files\\CDC - Zika - 02.16.16 | 36.48% | 47.15% | 16.38% | 0% | 16.38% | 83.63% |
| 18 : Files\\CDC - Zika - 02.19.16 | 0% | 0% | 0% | 0% | 0.00% | 0.00% |
| 19 : Files\\CDC - Zika - 02.19.16 (2) | 0% | 100% | 0% | 0% | 0.00% | 100.00% |
| 20 : Files\\CDC - Zika - 02.21.16 | 16.36% | 46.67% | 36.97% | 0% | 36.97% | 63.03% |
| 21 : Files\\CDC - Zika - 02.22.16 | 36.63% | 29.64% | 18.55% | 15.18% | 33.73% | 66.27% |
| 22 : Files\\CDC - Zika - 02.23.16 | 38.85% | 43.21% | 16.85% | 1.09% | 17.94% | 82.06% |
| 23 : Files\\CDC - Zika - 03.04.16 | 25.84% | 35.5% | 29.32% | 9.34% | 38.66% | 61.34% |
| 24 : Files\\CDC - Zika - 03.06.16 | 33.59% | 34.64% | 21.61% | 10.16% | 31.77% | 68.23% |
| 25 : Files\\CDC - Zika - 03.06.16 (2) | 50.74% | 2.94% | 46.32% | 0% | 46.32% | 53.68% |
| 26 : Files\\CDC - Zika - 03.12.16 | 0% | 55.56% | 0% | 44.44% | 44.44% | 55.56% |
| 27 : Files\\CDC - Zika - 03.14.16 | 0% | 100% | 0% | 0% | 0.00% | 100.00% |
| 28 : Files\\CDC - Zika - 03.24.16 | 12.22% | 76.02% | 5.43% | 6.33% | 11.76% | 88.24% |
| 29 : Files\\CDC - Zika - 03.25.16 | 55.19% | 0% | 39.15% | 5.66% | 44.81% | 55.19% |
| 30 : Files\\CDC - Zika - 04.01.16 | 27.36% | 29.42% | 32.02% | 11.2% | 43.22% | 56.78% |
| 31 : Files\\CDC - Zika - 04.08.16 | 58.27% | 11.7% | 6.36% | 23.66% | 30.02% | 69.97% |
| 32 : Files\\CDC - Zika - 04.11.16 | 33.35% | 50.77% | 12.25% | 3.63% | 15.88% | 84.12% |
| 33 : Files\\CDC - Zika - 04.13.16 | 29.52% | 37.02% | 26.55% | 6.91% | 33.46% | 66.54% |
| 34 : Files\\CDC - Zika - 04.19.16 | 14.65% | 71.66% | 0% | 13.69% | 13.69% | 86.31% |
| 35 : Files\\CDC - Zika - 04.20.16 | 15.49% | 41.75% | 28.28% | 14.48% | 42.76% | 57.24% |
| 36 : Files\\CDC - Zika - 04.27.16 | 0% | 0% | 100% | 0% | 100.00% | 0.00% |
| 37 : Files\\CDC - Zika - 05.13.15 | 50% | 20.53% | 29.47% | 0% | 29.47% | 70.53% |
| 38 : Files\\CDC - Zika - 05.15.16 | 46.21% | 34.48% | 10.29% | 9.03% | 19.32% | 80.69% |
| 39 : Files\\CDC - Zika - 05.20.16 | 0% | 0% | 0% | 0% | 0.00% | 0.00% |
| 40 : Files\\CDC - Zika - 05.22.15 | 59.52% | 0% | 0% | 40.48% | 40.48% | 59.52% |
| 41 : Files\\CDC - Zika - 05.25.16 | 0% | 0% | 0% | 0% | 0.00% | 0.00% |
| 42 : Files\\CDC - Zika - 05.31.16 | 0% | 0% | 0% | 100% | 100.00% | 0.00% |
| 43 : Files\\CDC - Zika - 06.03.16 | 0% | 48.06% | 48.06% | 3.88% | 51.94% | 48.06% |
| 44 : Files\\CDC - Zika - 06.07.16 | 24.54% | 48.71% | 8.85% | 17.9% | 26.75% | 73.25% |
| 45 : Files\\CDC - Zika - 06.08.16 | 21.13% | 34.39% | 32.63% | 11.85% | 44.48% | 55.52% |
| 46 : Files\\CDC - Zika - 06.08.16 (2) | 36.74% | 35.3% | 8.42% | 19.53% | 27.95% | 72.04% |
| 47 : Files\\CDC - Zika - 06.13.16 | 40.43% | 23.04% | 13.26% | 23.26% | 36.52% | 63.47% |
| 48 : Files\\CDC - Zika - 07.04.16 | 60.99% | 0% | 0% | 39.01% | 39.01% | 60.99% |
| 49 : Files\\CDC - Zika - 07.06.16 | 35.98% | 43.94% | 15.15% | 4.92% | 20.07% | 79.92% |
| 50 : Files\\CDC - Zika - 07.11.16 | 0% | 50% | 50% | 0% | 50.00% | 50.00% |
| 51 : Files\\CDC - Zika - 07.18.16 | 100% | 0% | 0% | 0% | 0.00% | 100.00% |
| 52 : Files\\CDC - Zika - 07.25.16 | 24.31% | 36.81% | 38.89% | 0% | 38.89% | 61.12% |
| 53 : Files\\CDC - Zika - 07.27.16 | 56.91% | 13.3% | 29.26% | 0.53% | 29.79% | 70.21% |
| 54 : Files\\CDC - Zika - 07.29.16 | 42.82% | 21.47% | 17.72% | 17.98% | 35.70% | 64.29% |
| 55 : Files\\CDC - Zika - 07.30.16 | 36.12% | 12.55% | 40.68% | 10.65% | 51.33% | 48.67% |
| 56 : Files\\CDC - Zika - 08.01.16 | 38.56% | 27.38% | 24.87% | 9.2% | 34.07% | 65.94% |
| 57 : Files\\CDC - Zika - 08.03.16 | 0% | 60% | 40% | 0% | 40.00% | 60.00% |
| 58 : Files\\CDC - Zika - 08.13.16 | 49.25% | 27.17% | 15.09% | 8.49% | 23.58% | 76.42% |
| 59 : Files\\CDC - Zika - 08.18.16 | 44.39% | 38.05% | 17.56% | 0% | 17.56% | 82.44% |
| 60 : Files\\CDC - Zika - 08.18.16 (2) | 33.81% | 32.62% | 25.3% | 8.27% | 33.57% | 66.43% |
| 61 : Files\\CDC - Zika - 08.19.16 | 30.41% | 56.7% | 12.89% | 0% | 12.89% | 87.11% |
| 62 : Files\\CDC - Zika - 08.23.16 | 31.23% | 35.49% | 20.82% | 12.46% | 33.28% | 66.72% |
| 63 : Files\\CDC - Zika - 08.25.16 | 0% | 0% | 0% | 100% | 100.00% | 0.00% |
| 64 : Files\\CDC - Zika - 08.26.16 | 0% | 0% | 0% | 0% | 0.00% | 0.00% |
| 65 : Files\\CDC - Zika - 08.27.16 | 42.09% | 21.41% | 30.9% | 5.6% | 36.50% | 63.50% |
| 66 : Files\\CDC - Zika - 08.30.16 | 69.59% | 30.41% | 0% | 0% | 0.00% | 100.00% |
| 67 : Files\\CDC - Zika - 08.31.16 | 29.77% | 43.59% | 12.23% | 14.41% | 26.64% | 73.36% |
| 68 : Files\\CDC - Zika - 09.09.16 | 38.98% | 22.04% | 12.25% | 26.73% | 38.98% | 61.02% |
| 69 : Files\\CDC - Zika - 09.11.16 | 68.91% | 23.08% | 0% | 8.01% | 8.01% | 91.99% |
| 70 : Files\\CDC - Zika - 09.14.16 | 100% | 0% | 0% | 0% | 0.00% | 100.00% |
| 71 : Files\\CDC - Zika - 09.15.16 | 86.92% | 0% | 13.08% | 0% | 13.08% | 86.92% |
| 72 : Files\\CDC - Zika - 09.19.16 | 19.51% | 43.75% | 36.74% | 0% | 36.74% | 63.26% |
| 73 : Files\\CDC - Zika - 09.22.16 | 0% | 43.9% | 56.1% | 0% | 56.10% | 43.90% |
| 74 : Files\\CDC - Zika - 09.22.16 (2) | 27.11% | 45.79% | 27.11% | 0% | 27.11% | 72.90% |
| 75 : Files\\CDC - Zika - 09.23.16 | 2.26% | 57.11% | 35.44% | 5.19% | 40.63% | 59.37% |
| 76 : Files\\CDC - Zika - 09.24.16 | 0% | 53.7% | 0% | 46.3% | 46.30% | 53.70% |
| 77 : Files\\CDC - Zika - 09.25.16 | 47.4% | 24.1% | 21.15% | 7.35% | 28.50% | 71.50% |
| 78 : Files\\CDC - Zika - 09.25.16 (2) | 54.23% | 28.36% | 10.95% | 6.47% | 17.42% | 82.59% |
| 79 : Files\\CDC - Zika - 09.26.16 | 48.71% | 28.02% | 23.28% | 0% | 23.28% | 76.73% |
| 80 : Files\\CDC - Zika - 10.11.16 | 29.95% | 14.98% | 41.01% | 14.06% | 55.07% | 44.93% |
| 81 : Files\\CDC - Zika - 10.13.16 | 35.81% | 24.45% | 21.83% | 17.9% | 39.73% | 60.26% |
| 82 : Files\\CDC - Zika - 10.18.16 | 29.52% | 46.1% | 11.58% | 12.79% | 24.37% | 75.62% |
| 83 : Files\\CDC - Zika - 10.19.16 | 51.36% | 25.91% | 0% | 22.73% | 22.73% | 77.27% |
| 84 : Files\\CDC - Zika - 11.12.16 | 22.94% | 46.79% | 21.1% | 9.17% | 30.27% | 69.73% |
| 85 : Files\\CDC - Zika - 11.28.16 | 29.68% | 37.9% | 20.97% | 11.45% | 32.42% | 67.58% |
| 86 : Files\\CDC - Zika - 12.15.16 | 39.15% | 32.95% | 3.88% | 24.03% | 27.91% | 72.10% |
| 87 : Files\\CDC - Zika - 12.19.16 | 18.41% | 57.01% | 18.72% | 5.87% | 24.59% | 75.42% |
| 88 : Files\\CDC - Zika - 12.22.16 | 29.4% | 47.54% | 19.34% | 3.72% | 23.06% | 76.94% |
| 89 : Files\\CDC - Zika - 12.29.16 | 19.03% | 53.8% | 19.39% | 7.78% | 27.17% | 72.83% |
| 90 : Files\\CDC - Zika - 12.29.16 (2) | 31.58% | 34.6% | 28.04% | 5.78% | 33.82% | 66.18% |
| 91 : Files\\FDA - Dengue - 08.17.16 | 42.84% | 25.08% | 27.52% | 4.55% | 32.07% | 67.92% |
| 92 : Files\\FDA - Dengue - 12.16.16 | 0% | 0% | 0% | 0% | 0.00% | 0.00% |
| 93 : Files\\FDA- Zika - 02.16.16 | 0% | 0% | 94.36% | 5.64% | 100.00% | 0.00% |
| 94 : Files\\FDA- Zika - 02.17.16 | 0% | 0% | 0% | 0% | 0.00% | 0.00% |
| 95 : Files\\FDA- Zika - 02.23.16 | 0% | 0% | 0% | 0% | 0.00% | 0.00% |
| 96 : Files\\FDA- Zika - 02.26.16 | 18.41% | 0% | 79.92% | 1.67% | 81.59% | 18.41% |
| 97 : Files\\FDA- Zika - 02.26.16 (2) | 41.75% | 38.66% | 19.59% | 0% | 19.59% | 80.41% |
| 98 : Files\\FDA- Zika - 03.02.16 | 0% | 0% | 100% | 0% | 100.00% | 0.00% |
| 99 : Files\\FDA- Zika - 03.30.16 | 16.45% | 31.6% | 15.58% | 36.36% | 51.94% | 48.05% |
| 100 : Files\\FDA- Zika - 04.04.16 | 30.33% | 29.51% | 40.16% | 0% | 40.16% | 59.84% |
| 101 : Files\\FDA- Zika - 04.06.16 | 100% | 0% | 0% | 0% | 0.00% | 100.00% |
| 102 : Files\\FDA- Zika - 04.29.16 | 24.39% | 51.63% | 0% | 23.98% | 23.98% | 76.02% |
| 103 : Files\\FDA- Zika - 05.16.16 | 0% | 0% | 100% | 0% | 100.00% | 0.00% |
| 104 : Files\\FDA- Zika - 06.13.16 | 43% | 0% | 13.99% | 43% | 56.99% | 43.00% |
| 105 : Files\\FDA- Zika - 06.17.16 | 0% | 0% | 100% | 0% | 100.00% | 0.00% |
| 106 : Files\\FDA- Zika - 06.21.16 | 0% | 0% | 0% | 0% | 0.00% | 0.00% |
| 107 : Files\\FDA- Zika - 06.21.16 (2) | 100% | 0% | 0% | 0% | 0.00% | 100.00% |
| 108 : Files\\FDA- Zika - 06.22.16 | 0% | 100% | 0% | 0% | 0.00% | 100.00% |
| 109 : Files\\FDA- Zika - 06.24.16 | 27.27% | 32.02% | 13.44% | 27.27% | 40.71% | 59.29% |
| 110 : Files\\FDA- Zika - 06.24.16 (2) | 0% | 100% | 0% | 0% | 0.00% | 100.00% |
| 111 : Files\\FDA- Zika - 06.27.16 | 18.66% | 52.24% | 29.1% | 0% | 29.10% | 70.90% |
| 112 : Files\\FDA- Zika - 06.29.16 | 100% | 0% | 0% | 0% | 0.00% | 100.00% |
| 113 : Files\\FDA- Zika - 07.05.16 | 0% | 0% | 0% | 0% | 0.00% | 0.00% |
| 114 : Files\\FDA- Zika - 07.12.16 | 0% | 100% | 0% | 0% | 0.00% | 100.00% |
| 115 : Files\\FDA- Zika - 07.12.16 picture | 0% | 0% | 0% | 0% | 0.00% | 0.00% |
| 116 : Files\\FDA- Zika - 07.20.16 | 0% | 0% | 19.4% | 80.6% | 100.00% | 0.00% |
| 117 : Files\\FDA- Zika - 07.21.16 | 53.57% | 16.23% | 17.53% | 12.66% | 30.19% | 69.80% |
| 118 : Files\\FDA- Zika - 07.22.16 | 100% | 0% | 0% | 0% | 0.00% | 100.00% |
| 119 : Files\\FDA- Zika - 07.26.16 | 0% | 100% | 0% | 0% | 0.00% | 100.00% |
| 120 : Files\\FDA- Zika - 07.28.16 | 0% | 0% | 100% | 0% | 100.00% | 0.00% |
| 121 : Files\\FDA- Zika - 07.29.16 | 34.21% | 0% | 0% | 65.79% | 65.79% | 34.21% |
| 122 : Files\\FDA- Zika - 07.29.16 (2) | 0% | 100% | 0% | 0% | 0.00% | 100.00% |
| 123 : Files\\FDA- Zika - 08.02.16 | 50.82% | 0% | 49.18% | 0% | 49.18% | 50.82% |
| 124 : Files\\FDA- Zika - 08.08.16 | 20.34% | 12.54% | 8.81% | 58.31% | 67.12% | 32.88% |
| 125 : Files\\FDA- Zika - 08.17.16 | 65.75% | 12.79% | 14.16% | 7.31% | 21.47% | 78.54% |
| 126 : Files\\FDA- Zika - 08.17.16 (2) | 44.84% | 24.19% | 26.56% | 4.41% | 30.97% | 69.03% |
| 127 : Files\\FDA- Zika - 08.18.16 | 0% | 65.43% | 34.57% | 0% | 34.57% | 65.43% |
| 128 : Files\\FDA- Zika - 08.19.16 | 1.47% | 98.53% | 0% | 0% | 0.00% | 100.00% |
| 129 : Files\\FDA- Zika - 08.19.16 (2) | 42.02% | 13.3% | 40.96% | 3.72% | 44.68% | 55.32% |
| 130 : Files\\FDA- Zika - 08.23.16 | 14.67% | 15.22% | 23.91% | 46.2% | 70.11% | 29.89% |
| 131 : Files\\FDA- Zika - 08.24.16 | 0% | 0% | 0% | 0% | 0.00% | 0.00% |
| 132 : Files\\FDA- Zika - 08.25.16 | 0% | 25% | 0% | 75% | 75.00% | 25.00% |
| 133 : Files\\FDA- Zika - 08.26.16 | 100% | 0% | 0% | 0% | 0.00% | 100.00% |
| 134 : Files\\FDA- Zika - 08.26.16 (2) | 51.91% | 39.34% | 8.74% | 0% | 8.74% | 91.25% |
| 135 : Files\\FDA- Zika - 08.26.16 (3) | 100% | 0% | 0% | 0% | 0.00% | 100.00% |
| 136 : Files\\FDA- Zika - 08.30.16 | 0% | 0% | 100% | 0% | 100.00% | 0.00% |
| 137 : Files\\FDA- Zika - 08.31.16 | 0% | 31.53% | 59.46% | 9.01% | 68.47% | 31.53% |
| 138 : Files\\FDA- Zika - 08.31.16 (2) | 16.99% | 58.17% | 16.99% | 7.84% | 24.83% | 75.16% |
| 139 : Files\\FDA- Zika - 09.02.16 | 47.26% | 28.86% | 23.88% | 0% | 23.88% | 76.12% |
| 140 : Files\\FDA- Zika - 09.03.16 | 39.1% | 49.73% | 11.17% | 0% | 11.17% | 88.83% |
| 141 : Files\\FDA- Zika - 09.07.16 | 0% | 0% | 0% | 0% | 0.00% | 0.00% |
| 142 : Files\\FDA- Zika - 09.09.16 | 0% | 100% | 0% | 0% | 0.00% | 100.00% |
| 143 : Files\\FDA- Zika - 09.12.16 | 0% | 31.6% | 64.42% | 3.99% | 68.41% | 31.60% |
| 144 : Files\\FDA- Zika - 09.14.16 | 80.65% | 0% | 19.35% | 0% | 19.35% | 80.65% |
| 145 : Files\\FDA- Zika - 09.16.16 | 0% | 0% | 0% | 0% | 0.00% | 0.00% |
| 146 : Files\\FDA- Zika - 09.17.16 | 10.15% | 36.09% | 36.09% | 17.67% | 53.76% | 46.24% |
| 147 : Files\\FDA- Zika - 09.22.16 | 27.17% | 0% | 23.7% | 49.13% | 72.83% | 27.17% |
| 148 : Files\\FDA- Zika - 09.23.16 | 0% | 100% | 0% | 0% | 0.00% | 100.00% |
| 149 : Files\\FDA- Zika - 09.26.16 | 73.17% | 17.07% | 9.76% | 0% | 9.76% | 90.24% |
| 150 : Files\\FDA- Zika - 10.04.16 | 18.9% | 39.63% | 29.66% | 11.81% | 41.47% | 58.53% |
| 151 : Files\\FDA- Zika - 10.07.16 | 30.86% | 21.3% | 47.84% | 0% | 47.84% | 52.16% |
| 152 : Files\\FDA- Zika - 10.14.16 | 0% | 61.11% | 38.89% | 0% | 38.89% | 61.11% |
| 153 : Files\\FDA- Zika - 10.17.16 | 0% | 100% | 0% | 0% | 0.00% | 100.00% |
| 154 : Files\\FDA- Zika - 11.01.16 | 19.03% | 29.55% | 44.41% | 7.01% | 51.42% | 48.58% |
| 155 : Files\\FDA- Zika - 11.04.16 | 0% | 0% | 0% | 0% | 0.00% | 0.00% |
| 156 : Files\\FDA- Zika - 11.08.16 | 0% | 0% | 0% | 0% | 0.00% | 0.00% |
| 157 : Files\\FDA- Zika - 11.17.16 | 0% | 0% | 0% | 0% | 0.00% | 0.00% |
| 158 : Files\\FDA- Zika - 11.22.16 | 20.34% | 28.97% | 29.31% | 21.38% | 50.69% | 49.31% |
| 159 : Files\\FDA- Zika - 11.25.16 | 44.89% | 14.67% | 6.67% | 33.78% | 40.45% | 59.56% |
| 160 : Files\\FDA- Zika - 11.29.16 | 0% | 0% | 0% | 0% | 0.00% | 0.00% |
| 161 : Files\\FDA- Zika - 11.30.16 | 0% | 53.85% | 46.15% | 0% | 46.15% | 53.85% |
| 162 : Files\\FDA- Zika - 12.02.16 | 52.76% | 34.65% | 12.6% | 0% | 12.60% | 87.41% |
| 163 : Files\\FDA- Zika - 12.06.16 | 0% | 50% | 50% | 0% | 50.00% | 50.00% |
| 164 : Files\\FDA- Zika - 12.08.16 | 66.67% | 0% | 0% | 33.33% | 33.33% | 66.67% |
| 165 : Files\\FDA- Zika - 12.09.16 | 0% | 0% | 0% | 100% | 100.00% | 0.00% |
| 166 : Files\\FDA- Zika - 12.13.16 | 0% | 100% | 0% | 0% | 0.00% | 100.00% |
| 167 : Files\\FDA- Zika - 12.16.16 | 0% | 0% | 100% | 0% | 100.00% | 0.00% |
| 168 : Files\\FDA- Zika - 12.20.16 | 44.62% | 39.78% | 9.68% | 5.91% | 15.59% | 84.40% |
| 169 : Files\\FDA- Zika - 12.26.16 | 0% | 35.56% | 0% | 64.44% | 64.44% | 35.56% |
| 170 : Files\\FDA- Zika - 12.29.16 | 0% | 0% | 0% | 0% | 0.00% | 0.00% |
| 171 : Files\\FDA- Zika - 12.30.16 | 0% | 0% | 0% | 0% | 0.00% | 0.00% |
| 172 : Files\\NIAID - Dengue - 01.14.16 | 0% | 16.87% | 80.72% | 2.41% | 83.13% | 16.87% |
| 173 : Files\\NIAID - Dengue - 03.16.16 | 30% | 22.63% | 47.37% | 0% | 47.37% | 52.63% |
| 174 : Files\\NIAID - Dengue - 08.16.16 | 0% | 100% | 0% | 0% | 0.00% | 100.00% |
| 175 : Files\\NIAID - Dengue - 08.17.16 | 21.59% | 12.5% | 0% | 65.91% | 65.91% | 34.09% |
| 176 : Files\\NIAID - Zika - 02.10.16 | 0% | 0% | 0% | 100% | 100.00% | 0.00% |
| 177 : Files\\NIAID - Zika - 03.31.16 | 46.43% | 0% | 53.57% | 0% | 53.57% | 46.43% |
| 178 : Files\\NIAID - Zika - 05.11.16 | 65.33% | 0% | 34.67% | 0% | 34.67% | 65.33% |
| 179 : Files\\NIAID - Zika - 05.17.16 | 0% | 0% | 0% | 0% | 0.00% | 0.00% |
| 180 : Files\\NIAID - Zika - 05.19.16 | 0% | 33.62% | 66.38% | 0% | 66.38% | 33.62% |
| 181 : Files\\NIAID - Zika - 06.21.16 | 0% | 100% | 0% | 0% | 0.00% | 100.00% |
| 182 : Files\\NIAID - Zika - 06.24.16 | 0% | 100% | 0% | 0% | 0.00% | 100.00% |
| 183 : Files\\NIAID - Zika - 06.28.16 | 0% | 0% | 0% | 0% | 0.00% | 0.00% |
| 184 : Files\\NIAID - Zika - 07.27.16 | 0% | 0% | 100% | 0% | 100.00% | 0.00% |
| 185 : Files\\NIAID - Zika - 08.04.16 | 0% | 0% | 100% | 0% | 100.00% | 0.00% |
| 186 : Files\\NIAID - Zika - 08.15.16 | 0% | 100% | 0% | 0% | 0.00% | 100.00% |
| 187 : Files\\NIAID - Zika - 08.15.16 (2) | 0% | 0% | 33.33% | 66.67% | 100.00% | 0.00% |
| 188 : Files\\NIAID - Zika - 08.15.16 (3) | 0% | 0% | 100% | 0% | 100.00% | 0.00% |
| 189 : Files\\NIAID - Zika - 08.15.16 (4) | 0% | 0% | 0% | 100% | 100.00% | 0.00% |
| 190 : Files\\NIAID - Zika - 08.16.16 | 23.7% | 28.91% | 26.55% | 20.84% | 47.39% | 52.61% |
| 191 : Files\\NIAID - Zika - 08.16.16 (2) | 0% | 100% | 0% | 0% | 0.00% | 100.00% |
| 192 : Files\\NIAID - Zika - 08.17.16 | 21.59% | 12.5% | 0% | 65.91% | 65.91% | 34.09% |
| 193 : Files\\NIAID - Zika - 09.22.16 | 0% | 0% | 100% | 0% | 100.00% | 0.00% |
| 194 : Files\\NIAID - Zika - 09.28.16 | 0% | 0% | 0% | 0% | 0.00% | 0.00% |
| 195 : Files\\NIAID - Zika - 11.04.16 | 50% | 0% | 0% | 50% | 50.00% | 50.00% |
| 196 : Files\\NIAID - Zika - 11.07.16 | 0% | 100% | 0% | 0% | 0.00% | 100.00% |
| 197 : Files\\NIAID - Zika - 11.07.16 (2) | 0% | 50% | 50% | 0% | 50.00% | 50.00% |
| 198 : Files\\NIAID - Zika - 11.17.16 | 28.7% | 42.59% | 0% | 28.7% | 28.70% | 71.29% |
| 199 : Files\\NIAID - Zika - 12.29.16 | 0% | 0% | 0% | 0% | 0.00% | 0.00% |
| 200 : Files\\NIH - Dengue - 03.16.16 | 14.47% | 21.7% | 16.6% | 47.23% | 63.83% | 36.17% |
| 201 : Files\\NIH - Zika - 01.25.16 | 48.48% | 51.52% | 0% | 0% | 0.00% | 100.00% |
| 202 : Files\\NIH - Zika - 03.15.16 | 0% | 100% | 0% | 0% | 0.00% | 100.00% |
| 203 : Files\\NIH - Zika - 04.23.16 | 0% | 0% | 0% | 0% | 0.00% | 0.00% |
| 204 : Files\\NIH - Zika - 05.05.16 | 19.94% | 21.78% | 44.48% | 13.8% | 58.28% | 41.72% |
| 205 : Files\\NIH - Zika - 05.19.16 | 50% | 0% | 0% | 50% | 50.00% | 50.00% |
| 206 : Files\\NIH - Zika - 06.10.16 | 0% | 0% | 0% | 0% | 0.00% | 0.00% |
| 207 : Files\\NIH - Zika - 06.28.16 | 0% | 0% | 100% | 0% | 100.00% | 0.00% |
| 208 : Files\\NIH - Zika - 06.28.16 (2) | 0% | 100% | 0% | 0% | 0.00% | 100.00% |
| 209 : Files\\NIH - Zika - 07.05.16 | 0% | 100% | 0% | 0% | 0.00% | 100.00% |
| 210 : Files\\NIH - Zika - 08.16.16 | 21.25% | 18.32% | 43.59% | 16.85% | 60.44% | 39.57% |
| 211 : Files\\NIH - Zika - 09.07.16 | 46.48% | 53.52% | 0% | 0% | 0.00% | 100.00% |
| 212 : Files\\NIH - Zika - 10.14.16 | 41.67% | 58.33% | 0% | 0% | 0.00% | 100.00% |
| 213 : Files\\NIH - Zika - 11.08.16 | 53.04% | 0% | 46.96% | 0% | 46.96% | 53.04% |
| 214 : Files\\NIH - Zika - 11.26.16 | 0% | 0% | 0% | 100% | 100.00% | 0.00% |
